# Which hand is mine? Discriminating body ownership perception in a two-alternative forced-choice task

**DOI:** 10.3758/s13414-020-02107-x

**Published:** 2020-08-27

**Authors:** Marie Chancel, H. Henrik Ehrsson

**Affiliations:** Department of Neuroscience, Brain, Body and Self Laboratory, Karolinska Institute, SE-171 77 Stockholm, Sweden

**Keywords:** Body ownership, Multisensory integration, Psychophysics, Rubber hand illusion, Body perception, Self-attribution

## Abstract

The experience of one’s body as one’s own is referred to as the sense of body ownership. This central part of human conscious experience determines the boundary between the self and the external environment, a crucial distinction in perception, action, and cognition. Although body ownership is known to involve the integration of signals from multiple sensory modalities, including vision, touch, and proprioception, little is known about the principles that determine this integration process, and the relationship between body ownership and perception is unclear. These uncertainties stem from the lack of a sensitive and rigorous method to quantify body ownership. Here, we describe a two-alternative forced-choice discrimination task that allows precise and direct measurement of body ownership as participants decide which of two rubber hands feels more like their own in a version of the rubber hand illusion. In two experiments, we show that the temporal and spatial congruence principles of multisensory stimulation, which determine ownership discrimination, impose tighter constraints than previously thought and that texture congruence constitutes an additional principle; these findings are compatible with theoretical models of multisensory integration. Taken together, our results suggest that body ownership constitutes a genuine perceptual multisensory phenomenon that can be quantified with psychophysics in discrimination experiments.

**Electronic supplementary material:**

The online version of this article (10.3758/s13414-020-02107-x) contains supplementary material, which is available to authorized users.

One’s body is the most important object in one’s life, serving as the essential medium through which one experiences the world and interacts with others and with one’s environment. Perceiving what constitutes this body (i.e., which objects are parts of one’s body and which are not) is therefore of crucial importance for the ability to act upon one’s surroundings as well as to protect one’s physical integrity. The experience of the body as one’s own is referred to as the sense (or feeling) of “body ownership” (Ehrsson, 2012). This experience includes the phenomenological quality that a body part is part of one’s body (Martin, 1995) and subjective awareness of a limb or the whole body as one’s own (Gallagher, 2000; Gallagher & Daly, 2018). As such, body ownership constitutes a fundamental component of self-awareness, a fact that has intrigued both scientists and philosophers (Merleau-Ponty, 1962). The complexity and the importance of body ownership have also been highlighted by case studies in clinical neurology, in which it was noted that people suffering from stroke, often involving the frontal and posterior parietal areas of the right hemisphere, can demonstrate inability to recognize limbs as part of their own body (asomatognosia and somatoparaphrenia; Arzy, Overney, Landis, & Blanke, 2006; Critchley, 1953; Feinberg, Venneri, Simone, Fan, & Northoff, 2010). The existence of these neurological cases suggests that body ownership is a neurocognitive function that can be selectively impaired and points towards the associative cortex as the neural substrate, as the primary sensory areas and basic sensory capacities are sometimes intact in these individuals. However, these clinical observations do not provide information on the specific cognitive processes and brain mechanisms involved in body ownership, as the lesions are large and typically damage many different areas as well as white matter fiber tracts that connect widespread regions.

Twenty years ago, Botvinick and Cohen (1998) published a ground-breaking study on healthy participants that sparked the modern interest in experimental studies of body ownership (see also Tastevin, 1937). In this study, the investigators described what is known as the rubber hand illusion (RHI): When participants watched a life-sized rubber hand being stroked in the same way and at the same time as strokes were delivered to their real hand, which was hidden behind a screen, they began to feel that the rubber hand was their own hand and that it sensed the touches of the paintbrush. This illusion highlights the remarkable flexibility of body representation and provides scientists with a very useful experimental model to investigate the sense of body ownership. The synchrony of the visuotactile stimulation (Botvinick & Cohen, 1998; Shimada, Fukuda, & Hiraki, 2009), the visual resemblance between the rubber hand and a human hand (Tsakiris, Carpenter, James, & Fotopoulou, 2010), the postural correspondence between the rubber hand and the real hand (Ehrsson, Spence, & Passingham, 2004; Pavani, Spence, & Driver, 2000), and the spatial proximity of the rubber hand to the real hand (Kalckert & Ehrsson, 2014; Lloyd, 2007; Preston, 2013) all provide the brain with sensory evidence in favor of the conclusion that the rubber hand is one’s own hand (for a recent review, see Kilteni, Maselli, Kording, & Slater, 2015). If the touches applied to the rubber hand and the real hand are asynchronous or the model hand is presented in an anatomically impossible orientation or placed further than approximately 30 cm from the real hand, the illusion is eliminated or significantly reduced (Ehrsson et al., 2004; Guterstam, Gentile, & Ehrsson, 2013; Haans, Ijsselsteijn, & de Kort, 2008; Romano, Caffa, Hernandez-Arieta, Brugger, & Maravita, 2015; Tsakiris et al., 2010). These spatial and temporal constraints of the illusion are reminiscent of the temporal and spatial principles of multisensory integration (Holmes & Spence, 2005; Makin, Holmes, & Ehrsson, 2008; Stein & Stanford, 2008; for a complementary discussion on specific tasks and contexts in which the spatial congruence do not influence multisensory integration see Spence, 2013); therefore, the RHI is often referred to as a “multisensory illusion” (Ehrsson, 2012; Kilteni et al., 2015; Samad, Chung, & Shams, 2015). However, the exact relationship between body ownership and multisensory perception is not fully understood and remains a matter of ongoing debate in the literature.

This controversy stems from a fundamental question: Exactly what type of psychological phenomenon does body ownership constitute? On what kinds of processes does body ownership depend? Does this phenomenon constitute a perception or some form of higher cognitive function? In the literature, body ownership is often referred to as a “feeling” of ownership (Ehrsson et al., 2004; Tsakiris, 2017) or a core element of “bodily self-consciousness” (Blanke, Ionta, Fornari, Mohr, & Maeder, 2010; Blanke, Slater, & Serino, 2015), but it is sometimes also merged into broader concepts such as the “bodily self” (Bermúdez, 1998; Legrand, 2006; Riemer et al., 2014) or “embodiment” (i.e., the experience of having a body and controlling it; Arzy et al., 2006; Bassolino et al., 2018; Longo, Schüür, Kammers, Tsakiris, & Haggard, 2008). Body ownership has also been referred to as a “phenomenological sensation of incorporation” (Schütz-Bosbach, Tausche, & Weiss, 2009), and it has been called an “ability to know [which body parts belong to us]” (Butler, Héroux, & Gandevia, 2017) or used interchangeably with the term “corporeal awareness” (Berlucchi & Aglioti, 1997). Longo’s thought summarizes this issue well: “It is clearly a kind of experience, but psychology’s traditional methods of studying experience have difficulty in capturing its nature” (Longo et al., 2008). Although many scientists in the field agree that interactions between different sensory modalities, including vision, touch, and limb position sense (proprioception), play an important role in body ownership, the exact relationship between body ownership and multisensory integration is not clear. Can body ownership be equated with multisensory perception of one’s body (Ehrsson, 2012), or is multisensory integration merely one process among several that make up body ownership in a more complex cognitive architecture (Tsakiris et al., 2010)? Alternatively, is body ownership a higher-order cognitive process distinct from multisensory perception, such as conceptual knowledge about oneself as a person or recognition memory of familiar stimuli? Considering these open questions, pinpointing what type of process body ownership constitutes and clarifying its relation to multisensory perception would constitute a major conceptual advance in the field. However, as Longo et al. (2008) concludes, the field needs new methods that measure body ownership more precisely and rigorously than existing methods can.

Indeed, a major issue in body ownership research is the lack of a quantitative approach to directly and exactly measure the phenomenon of interest. To date, researchers have used combinations of different subjective and indirect objective tests. In the previous literature, the most common method to assess the sense of body ownership is the use of questionnaires (Botvinick & Cohen, 1998; Longo et al., 2008). Participants are asked, on different items, to rate how much they subjectively felt that the (rubber) hand they saw was their own. Questionnaires are easy to administer, and they can directly assess body ownership, but, unfortunately, their reliability is questionable (Hoskin, 2012). First, even when acting in good faith on careful instructions from the experimenter, participants may not be aware of subtle changes in their perception (poor introspective ability). Second, questionnaires are susceptible to cognitive bias and task compliance effects (i.e., the participants want to help the experimenter), and this desire affects the questionnaire results independently of genuine perception. Third, the questionnaires are typically administered *after* the induction of the illusions, which means that the participants must rely on memory (but see Kokkinara & Slater, 2019, for a method to probe changes in ownership concurrent with the illusion). In the memory process, subtle nuances between illusion conditions may be lost, and the memory trace of the original perceptual experience can be influenced by various postperceptual cognitive processes. Finally, participants use different strategies to fill out the scales (Austin, Deary, Gibson, McGregor, & Dent, 1998; Balakrishnan, 1999). For all the reasons mentioned above, questionnaires alone are insufficient to address fundamental questions related to perception, especially if the perceptual effects of interest are small and appropriate control conditions are not included.

Because of these problems, questionnaires are often combined with various indirect objective measures of body ownership, but these measures come with their own limitations. The most commonly used indirect measure—known as “proprioceptive drift” (Botvinick & Cohen, 1998; Tsakiris & Haggard, 2005)—employs the perceived position of the real hand as an index of body ownership: With their eyes closed, participants are asked to point to or verbally report the location of their unseen (stimulated) hand, a procedure that is repeated before and after the induction of the RHI. After experiencing the illusion, the participants perceive their hand as being significantly closer to the rubber hand than before the illusion was induced, and the difference corresponds to proprioceptive drift. However, recent studies have questioned the validity of proprioceptive drift as an objective measure, raising doubt about its causal relationship to body ownership. For example, Rohde, Luca, and Ernst (2011) have observed a significant proprioceptive drift towards the rubber hand in a control condition where ownership for this rubber hand was unlikely to arise (i.e., when subjects merely looked at the model hand without any synchronous seen and felt strokes being applied; see also Holmes, Snijders, & Spence, 2006); furthermore, Abdulkarim and Ehrsson (2016) reported that subjectively experienced body ownership does not change when changes in hand position sense are introduced in the RHI paradigm, which suggests that a bidirectional causal relationship between proprioceptive drift and body ownership is unlikely. Thus, the central processes supporting body ownership and proprioceptive drift are probably different, making proprioceptive drift an indirect and nonideal measurement of body ownership.

Another often-used objective test of body ownership is the skin conductance response (SCR) evoked by physical threats applied to the rubber hand (e.g., sticking the rubber hand with a needle; Petkova & Ehrsson, 2009), “cutting” it with a knife (Guterstam, Petkova, & Ehrsson, 2011), or bending the rubber finger backward (Armel & Ramachandran, 2003). Physical threats directed at the body produce an emotional response (Ehrsson, Wiech, Weiskopf, Dolan, & Passingham, 2007) that leads to activation of the autonomic nervous system, which, in turn, produces increased sweating from the skin and thereby changes the conductance registered by surface electrodes. Nevertheless, several concerns must be raised, including the lack of specificity of the SCR. Many psychological states other than fear or anticipation of pain can trigger an SCR, such as surprise, amusement, and cognitive demand (Andreassi, 2013; Nagai, Critchley, Featherstone, Trimble, & Dolan, 2004). Thus, interfering thoughts with emotional content or changes in the participants’ state of attention can influence SCR results. Hence, SCR experiments require very well-matched control conditions to produce interpretable results. In addition to this methodological concern, the SCR to repeated presentation of threat stimuli decreases with time (Andreassi, 2013), limiting the number of trials that can be acquired for each participant, which, in turn, limits the number of experimental conditions that can be tested. Finally, people vary greatly in their basic skin conductance responsiveness, with some individuals showing no response at all, and this high interindividual variability prevents SCR from being a sensitive measure of body ownership at the group level. Other indirect measures of body ownership have also been proposed, such as the crossmodal congruency task (Pavani et al., 2000; Zopf, Savage, & Williams, 2010) and registering changes in the skin temperature of the hidden real hand (Moseley et al., 2008). However, the former is limited because the crossmodal stimuli used in the task may interfere with the body illusion under investigation and because the measure itself registers implicit changes in peri-personal space (Spence, Pavani, & Driver, 2004) rather than ownership experiences directly (for discussions of the relationship between peri-personal space and body ownership, see Brozzoli, Ehrsson, & Farnè, 2014; Serino, 2019); the problem with the latter is that the changes in skin temperature have been difficult to reproduce (de Haan et al., 2017; Rohde, Wold, Karnath, & Ernst, 2013), making this measure unreliable and controversial.

The objectives of the present study stem from the abovementioned methodological shortcomings and conceptual ambiguities regarding body ownership. The first aim is to create a new psychophysical paradigm that allows reliable assessment of the participant’s ability to discriminate body ownership in the RHI. Such approach would more directly measure body ownership than the indirect tests of proprioceptive drift and threat-evoked SCR, and the results would be less susceptible to various forms of cognitive bias than questionnaires. The second aim is to use this psychophysics task to examine the hypothesis that body ownership can be defined as multisensory perception of one’s own body. Since the time of its pioneering theorists at the end of the 19th century, psychophysics has carried both a theoretical framework and an efficient set of methodological tools to investigate human perceptual changes elicited by manipulating the intensity of a physical stimulus. Among the many interesting aspect of these techniques, one fundamental strength of psychophysics is the robustness of the obtained measures that emerge from the large number of repetitions of each experimental condition. Thus, we will employ this tool to investigate whether perception of hand ownership can be fitted to psychometric curves when systematic temporal and spatial incongruencies are introduced in visual and tactile signals. Finally, the third aim is to take advantage of the methodological strengths of our new method and put it to the test to an unresolved issue in the rubber hand illusion literature: the hypothesized effect of tactile congruence on body ownership (see further below). In summary, we aim to both introduce a new rigorous and presumably sensitive way to register body ownership and advance our conceptual knowledge of the processes that underpin this psychological phenomenon.

To this end, we created a paradigm consisting of a two-alternative forced-choice (2-AFC) discrimination task between two visually presented rubber hands, where the participant was required to decide which of the two rubber hands felt more like his or her own hand. We reasoned that developing a discrimination task that seeks to determine at what point the difference between the stimuli applied to the two rubber hands leads to a detectable change in perceived hand ownership, would be a particularly useful new method as it would allow the utilization of a number of traditional psychophysics measures to the problem of body ownership. Noteworthy, the discrimination approach we are proposing relies on relatively recent developments in body representation research where it has been shown that people can readily experience “three-hand versions” of the original RHI paradigm (Ehrsson, 2009; Fan & Ehrsson, 2020; Newport, Pearce, & Preston, 2010; Guterstam et al., 2011). In this version, synchronous strokes applied to two identical, visible rubber hands, and the participant’s hidden hand produced an illusory feeling that both rubber hands were one’s own (Ehrsson, 2009; Fan & Ehrsson, 2020). Moreover, synchronous visuotactile stimulation applied to one of the two rubber hands coupled with asynchronous stimulation to the other model hand leads to selective ownership for the synchronously stimulated model hand (Fan & Ehrsson, 2020). We reasoned that we could readily develop this setup into a 2-AFC psychophysical discrimination task for limb ownership by finely and systematically varying the degree of asynchrony in the visuotactile stimulation of one rubber hand with respect to the other rubber hand and the hidden real hand. Using this approach, we designed two experiments. In Experiment 1 and the associated control experiment, we tested the selectivity and specificity of our discrimination task with respect to the temporal and spatial rules of body ownership (Ehrsson, 2012). Thus, we hypothesized that the psychometric curves should reflect the precise manipulation of the degree of synchrony between the visual and tactile stimuli applied to the rubber hands and real hand, but only when the rubber hand is presented in an anatomically plausible position (Ehrsson et al., 2004). Moreover, we hypothesized that a relatively small manipulation of the distance between the rubber hands and the participant’s real hand—5 cm—should impact the perceptual decisions regarding body ownership. Earlier studies using traditional measures of the RHI found a significant reduction in illusion strength only for distances of approximately 10 cm or greater between the rubber and the real hand in paradigms where the distance between the real hand and the rubber hand was varied in steps of a minimum of 10 cm (Kalckert & Ehrsson, 2014; Kalckert, Perera, Ganesan, & Tan, 2019; Lloyd, 2007; Preston, 2013). This finding was interpreted as meaning that peri-personal space around the hand (peri-hand space; Brozzoli, Ehrsson, & Farnè, 2014) constituted a basic constraint of the illusion, as this space extends approximately 30 cm from the hand (Fogassi et al., 1996; Graziano, Hu, & Gross, 1997; Guterstam, Zeberg, Özçiftci, & Ehrsson, 2016). However, a strong prediction by the multisensory hypothesis of body ownership is that even smaller spatial incongruencies within peri-hand space should affect the sensation of ownership (Samad et al., 2015), given that even subtle spatial mismatches among visual, tactile, and proprioceptive signals should impair the integration process according to general models of multisensory perception (Ernst & Banks, 2002; Körding et al., 2007; van Beers, Wolpert, & Haggard, 2002).

In Experiment 2, we further assessed the usefulness and presumed increased sensitivity of our psychophysical discrimination method by addressing an unresolved issue in the previous literature. According to a multisensory integration framework for body ownership, an incongruency between the tactile properties of the objects touching the visible rubber hand and the hidden real hand should result in weakened visuotactile integration and a weakened RHI. The reason for this predicted effect is because such tactile incongruency violates the multisensory congruency (Stein, 2012) between the material properties of the seen and felt objects and thereby also breaks the “unity assumption” principle, which states that only meaningful combinations of crossmodal stimuli are integrated (De Gelder & Bertelson, 2003; Vatakis & Spence, 2007). However, in contrast to this prediction, Schütz-Bosbach et al. (2009) found no significant difference when comparing the RHI induced by congruent and incongruent objects touching the real hand and the rubber hand (soft cotton vs. rough sponge). A similar negative finding was reported in a more recent study, which found that relatively subtle incongruencies in the tactile properties of the seen and felt objects (smooth vs. rough brushes) did not significantly impact the RHI, although greater incongruencies did impair the illusion (pencil vs. paintbrush; Ward, Mensah, & Jünemann, 2015). We reasoned that if body ownership depends on multisensory integration, and our new psychophysics paradigm is sensitive enough, then it should be able to capture subtle changes in the participants’ discrimination of body ownership, even for small tactile incongruencies where both Schütz-Bosbach et al. (2009) and Ward et al. (2015) failed to detect an effect.

## Experiment 1

### Method

#### Participants

We recruited 30 healthy, naïve participants for the first experiment (14 females, ages 29.7 ± 7 years). We did not perform a power analysis because the proposed paradigm was new and we therefore had no information about effect sizes from earlier studies. Instead, we chose the sample size to match a typical sample size from previous RHI experiments in our research team, as well as other teams (e.g., Brozzoli et al., 2012; Guterstam et al., 2011; Preston, 2013; Rohde et al., 2011). Eleven additional participants (five males, ages 28.4 ± 3 years) were involved in the control experiment. This control experiment was conducted in smaller groups of participants because we simply wanted to verify that the ownership discriminations would break down at the single subject level—that is, not fit the Gaussian cumulative model and/or result in behaviorally nonsensical results (e.g., random guessing), by the control manipulations we were administering (see below). All volunteers provided their written informed consent prior to their participation. Each participant received 150 SEK as a compensation for each hour spent on the experiment. All experiments were approved by the Regional Ethical Review Board of Stockholm (since 2019 the Swedish Ethical Review Authority; application number 2018/471-31/2).

#### Inclusion test

In the main experiment, we asked participants to judge the relative degree of ownership they felt towards the two simultaneously presented rubber hands. To make meaningful such discriminations, we reasoned that it was necessary for the participants to be able to experience the basic RHI (involving only one rubber hand). However, we know that approximately 20%–25% of typical participants do not experience a reliable RHI (Kalckert & Ehrsson, 2014), and the reason for this probably relates to individual differences in how multisensory signals from the hand are combined (for example, a higher relative weighting on proprioception than vision in the integration process would work against the illusion and vice versa; Fan & Ehrsson, 2020). Thus, in line with common practice in the body representation literature for studies where the aim of the study requires the participants to experience the RHI, we only included participants that were able to experience the illusion (e.g., Ehrsson et al., 2004; Lloyd, 2007; Mohan et al., 2012; Nitta, Tomita, Zhang, Zhou, & Yamada, 2018; Tsakiris, Hesse, Boy, Haggard, & Fink, 2007; Wold, Limanowski, Walter, & Blankenburg, 2014). Thus, all participants were first tested in the classical RHI paradigm, and individuals that denied the RHI in this initial test were not included in the main discrimination experiment (see Supplementary Figures S.8 and S.9 for 2-AFC data collected from 15 volunteers who did not experience the RHI).

For this inclusion test, each participant sat with his or her right hand resting on a support beneath a small table. On this table, 15 cm above the hidden real hand, the participant viewed a life-sized cosmetic prosthetic male right hand (model 30916-R, Fillauer^®^, filled with plaster; a “rubber hand”) placed in the same orientation as the real hand. The participant kept his or her eyes fixed on the rubber hand while the experimenter used two small probes (firm plastic tubes, diameter: 7 mm) to stroke the rubber hand and the participant’s hidden hand for 12 s, synchronizing the timing of the stroking as closely as possible. Each stroke lasted for 1 s and extended approximately 1 cm; the strokes were applied to five different places along the real and rubber index fingers, at a frequency of 0.5 Hz. The characteristics of the strokes and the duration of the stimulation were designed to resemble the stimulation later applied by the robot during the discrimination task (see below). Then, the participant completed a questionnaire identical to the one used by Botvinick and Cohen (1998; see Supplementary Material). This questionnaire includes three items assessing ownership and four control items to be rated between −3 (*I completely disagree with this item*) and 3 (*I completely agree with this item*). Our inclusion criteria for a strong enough RHI to participate in the main psychophysics experiment were as follows: (a) the mean score on the ownership statements (Q1, Q2, Q3; see Table S.1 in the Supplementary Material) is greater than one, and (b) the difference between the mean score on the ownership items and the mean score on the control items is greater than one. Six participants (two females) did not reach this threshold; therefore, 24 subjects participated in the main experiment (see Table S.1 in the Supplementary Material). After this inclusion phase, the participants were introduced to the setup for the main experiment, and the task was explained to them. This pretesting session lasted 30 minutes. The same pretesting procedure and inclusion criteria was used for the control experiment. One female participant did not reach the inclusion criteria; therefore 10 participants were included in the control experiment.

#### Experimental setup (Fig. 1a)

In this setup, the participant’s right hand lay hidden, palm down, on a flat support surface beneath a table (30 cm lateral to the body midline), while, on this table (15 cm above the real hand), two identical right rubber hands were placed parallel to each other in anatomically plausible positions at the same distance from the real hand. This setup was chosen because it allows the RHI to be elicited for the left rubber hand only, the right rubber hand only, or for both rubber hands simultaneously, given the specific pattern of synchronous visuotactile stimulation (Ehrsson 2009; Fan & Ehrsson, 2020; see further below). Halfway between the two rubber hands, a white circular marker was positioned to serve as a fixation point for the participants. This fixation point was used to ensure that the participant did not look at one of the rubber hands more often than the other during the illusion experiments. The participant’s left hand rested on his or her lap. A chin rest and an elbow rest (Ergorest Oy^®^, Finland) ensured that the participant’s head and arm remained in a steady and relaxed posture throughout the experiments. Three robot arms (designed in our laboratory by Martti Mercurio and Marie Chancel; see Fig. S1 in the Supplementary Material) applied tactile stimuli (taps) to the index fingers of the two rubber hands and to the participant’s hidden real index finger. Each robot arm was made of three parts: two 17-cm-long, 3-cm-wide metal pieces and a metal slab (10 × 20 cm) as a support. The joint between the two metal pieces and the one between the proximal piece and the support were powered by two HS-7950TH Ultra Torque servos that included 7.4 V optimized coreless motors (Hitec Multiplex^®^, USA). The distal metal piece ended with a ring containing a plastic tube (diameter: 7 mm) that we used to touch the rubber hands and the participant’s real hand. Attached to the ends of those plastic tubes were E3X-HD41 fiber sensors (OMRON^®^, Netherlands) that allowed us to register the exact timing of the touch, since this device emits a red laser light and measures whether the light bounces back, the latter of which happens when the robots’ endings are in contact with the surface of the hands. These light detectors thus allowed us to monitor the intrinsic delay of our robotic system and ensure that it did not vary excessively from the theoretical values we aimed for in our experimental manipulation of visuotactile asynchrony. During the experiment, the participants wore earphones playing white noise to block any auditory information from the robots’ movements. The noise volume was adapted to each participant so that the sounds produced by the robot system could not be heard, and at the same time the volume was comfortable for the duration of the experiment.

**Fig. 1:**
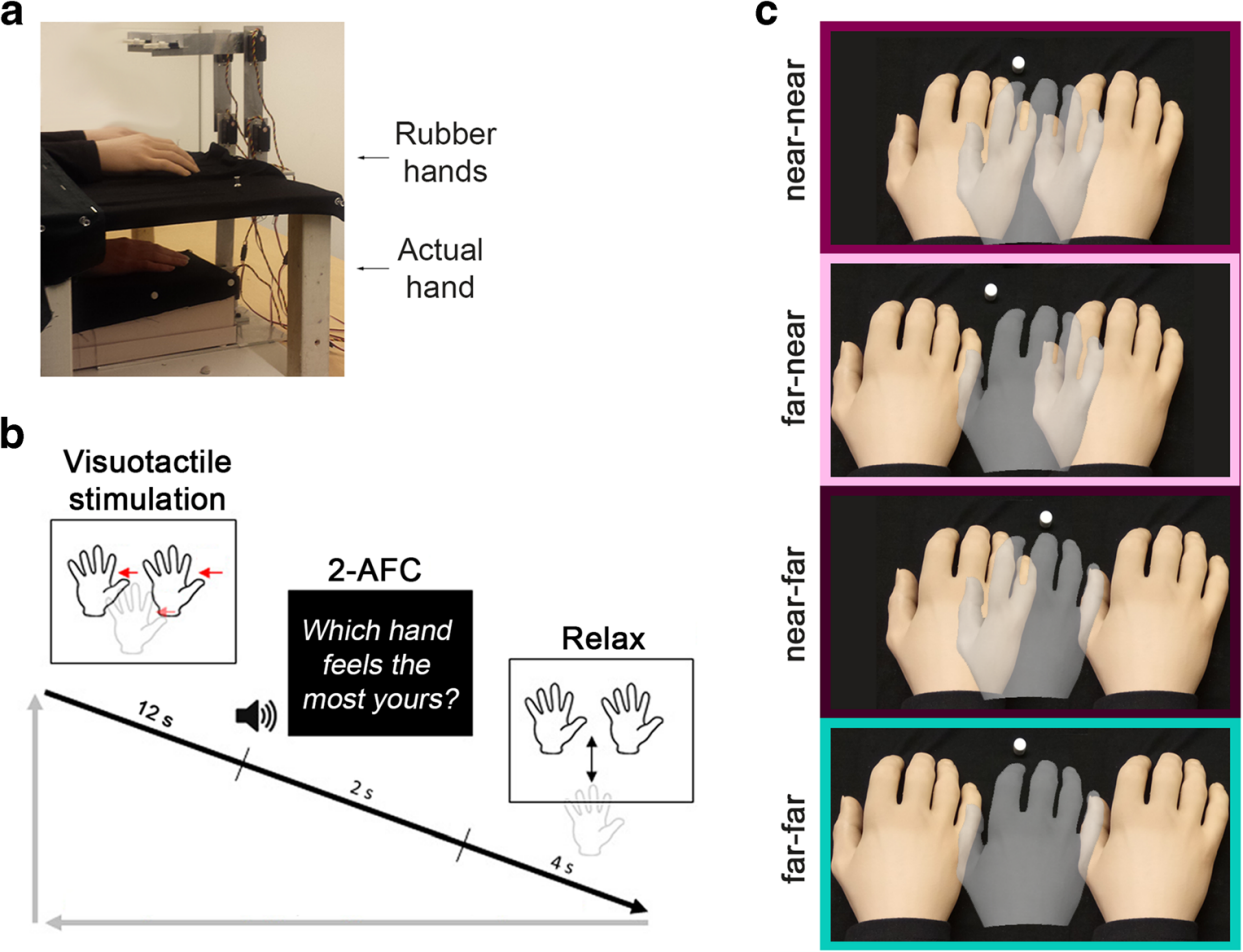
Experimental setup (**a**) and paradigm (**b, c**). A participant’s real right hand (semitransparent gray) is hidden under a table while they see two identical life-sized cosmetic prosthetic right hands (rubber hands; light skin color) on this table (**a**). The rubber hands and the real hand are touched for periods of 12 s, either synchronously or with one of the rubber hands touched slightly later at a degree of asynchrony that is systematically manipulated (50 ms, 100 ms, or 200 ms). The participant is then required to choose which of the two rubber hands feels more like his or her own in a 2-AFC (two-alternative forced choice) task (**b**). Four distance conditions are tested (**c**): near-near: Both rubber hands are positioned at the same distance from the real hand (5 cm); far-near: The right rubber hand (rRH) is closer (5 cm) to the real hand and the left rubber hand (lRH) is farther away (10 cm); near-far: The lRH is closer (5 cm) to the real hand, and the rRH is farther away (10 cm); far-far: Both rubber hands are placed at the same relatively far distance from the real hand (10 cm). The white dot halfway between the rubber hand indicates the fixation point

#### Procedure (Fig. 1b–c)

The task used in this experiment was a 2-AFC discrimination task. For each trial, participants were asked to choose which of the rubber hands felt most like their own hand (i.e., the one for which they had the strongest ownership illusion). Each trial followed the same sequence: The robots repeatedly tapped the index fingers of the rubber hands and the actual hand six times each for a total period of 12 s in five different locations in a randomized order (“stimulation phase”): just proximal to the nail on the distal phalanx, on the distal interphalangeal joint, on the middle phalanx, on the proximal interphalangeal joint, and on the proximal phalanx. Then, the robots stopped while the participants heard a tone instructing them to verbally report whether the *left* or the *right* rubber hand felt the most like their own (by saying “left” or “right”). A period of 12 s was chosen because earlier studies with individuals susceptible to the illusion have shown that the illusion is reliably elicited in approximately 10 s (Ehrsson et al., 2004; Guterstam et al., 2013; Lloyd, 2007). Different locations on the finger were chosen to avoid irritation of the skin during the long psychophysics session and were in line with earlier studies that often stimulate different parts of the hand and fingers to elicit the RHI (e.g., Guterstam et al., 2011). Informal pilots conducted on the authors and colleagues before the study commenced confirmed the RHI was elicited in our setup for the rubber hand that received the 12-s synchronous visuotactile stimulation.

After the stimulation period, the participants were asked to wiggle their right fingers to avoid any potential numbness or muscle stiffness from keeping their hand still and to eliminate possible carryover effect on the next stimulation period by breaking the illusory ownership sensations (the movement of the real hand while the rubber hand stays still abolishes the RHI). They also relaxed their gaze (i.e., they looked away from the fixation point and the rubber hands). Five seconds later, a second tone informed them that the next trial was about to start; the trial started 1 s after this sound cue. Every five trials, before that trial commenced, the participant was reminded to look at the white fixation marker placed halfway between the rubber hands during the stimulation phase.

Two variables are manipulated in this experiment: (1) the synchrony between the taps that are seen and the ones that are felt by the participants (= *asynchrony* conditions); and (2) the distance between the rubber hands and the participants’ actual hand on a lateral axis (= *distance* conditions). As described in the introduction, these two variables correspond to the temporal and spatial congruency principles of multisensory integration (Stein & Stanford, 2008). Thus, we predicted that the greater the congruency along these two dimensions, the stronger the sense of body ownership would be for a given rubber hand.

1. The protocol included seven *asynchronies*. The touches applied to the left or right rubber hand could be synchronized with the touches on the participant’s real hand, or they could be delayed by 50, 100, or 200 ms. For each trial, the touches on the participant’s real hand were synchronized with the touches on at least one of the rubber hands. For the rest of this article, the asynchronies on the right rubber hand (rRH) will be denoted as negative values (−50, −100, −200 ms), and those on the left rubber hand (lRH) as positive values (+50, +100, +200 ms). This positive/negative attribute indicates only which rubber hand is affected by a given asynchrony (i.e., how many milliseconds later this rubber hand is touched compared with the other rubber hand and the real hand). Note that in a more classic RHI paradigm, with just one rubber hand, all these magnitudes of visuotactile asynchrony should allow the emergence of the RHI (Shimada et al., 2009; Shimada, Suzuki, Yoda, & Hayashi, 2014).
2. Four different *distance* conditions were tested. In the baseline condition (near-near), the index finger of the left and right rubber hands are placed 5 cm to the left and right of the participants’ real index finger (perpendicular projection on the horizontal plan), respectively. In other conditions, one or both rubber hands are moved 5 cm further away from the real hand: either the lRH (condition far-near), the rRH (condition near-far) or both rubber hands (condition far-far). In the near-near and far-far conditions, both rubber hands are at the same distance from the real hand (approximately 18.0 cm and 15.8 cm, respectively, in three-dimensional space). Therefore, when both were touched at the same time as the real hand, we expected them to be equally likely to be chosen by the participants. In contrast, when one rubber hand is placed further away, we expect the closer rubber hand to be chosen more often as one’s own in line with the spatial congruency principle of multisensory integration.

Participants underwent four experimental blocks, one for each distance condition, each lasting 27 minutes, with the order of the blocks being counterbalanced between the participants. Within each block, the different asynchronies were presented in random order, with each of the seven different asynchronies repeated 12 times (−200 ms, 100 ms, 50 ms, 0 ms, +50 ms, +100 ms, and +200 ms). Thus, in each block, participants made 84 judgments of ownership between the rRH and the lRH.

#### Data analysis

To evaluate and compare participants’ perception across the four distance conditions (near-near, far-near, near-far, far-far), the observed data (i.e., the proportion of “the right hand feels the most like my hand” answers at different asynchronies) were fitted by the following cumulative Gaussian distribution:

$P(x)=\lambda +\left(1-2\lambda \right)\frac{1}{\sigma \sqrt{2\pi }}{\int}_{-\infty}^x{e}^{\frac{{\left(y-\mu \right)}^2}{2{\sigma}^2}} dy,$ (1)where *P*(*x*) is the probability of answering “right rubber hand” given the asynchrony *x, x* is the asynchrony between the touch that is felt and the one that it seen on the rubber hands (in ms), μ is the mean of the Gaussian (i.e., the point of subjective equality [PSE] that corresponds to the asynchrony leading the participant to perceive equal ownership for both rubber hands), and *σ* is the standard deviation (*SD*) of the curve, or discrimination threshold, which is inversely related to the participant’s discrimination sensitivity. In other words, a smaller *σ* value corresponds to a higher sensitivity to the asynchrony changes in the discrimination task. The indices PSE and *σ* characterize the participant’s body ownership. The lapse rate is *λ* : It accounts for stimulus-independent errors due to participants’ lapses (e.g., errors related to lack of focus, confusion between responses, response oversight). Our task was relatively easy, and the experiment was not too long and included regular breaks. Therefore, this lapse rate was restricted to small values, as it often is when psychophysical fitting procedures are designed ([0:0.06], Wichmann & Hill, 2001a, 2001b). This parameter does not provide information about the perceptual decision; thus, we disregarded it for the following analyses. The Palamedes toolbox implemented in MATLAB software (The MathWorks, Natick, MA) was used to fit the psychometric curves (Kingdom & Prins, 2009). With this toolbox, the goodness of fit of each participant’s responses by the chosen model (cumulative Gaussian) was assessed. When fitting our data to a cumulative Gaussian distribution, we assume that the probability of choosing the right rubber hand for one given asynchrony is related to the probability of choosing the right rubber hand for the other asynchronies, this association of probabilities being determined by the distribution we choose. For example, if a participant more often chooses the lRH as his or her own for one tested asynchrony, increasing the asynchrony on the rRH should lead to an even higher probability of selecting the lRH. Estimating the goodness of fit means testing this assumption against a random model in which the probability to choose the right rubber hand for one given asynchrony is not conditioned by the probability at the other asynchronies. The likelihood ratios of both those models are calculated over 1,500 simulations (bootstrap analysis) and compared with the ones obtained with our original experimental data using the Palamedes toolbox with the parameters recommended by its creators (Prins & Kingdom, 2018). We obtain the critical value *p*DEV (*p* value for deviance; i.e., the statistical value comparing the likelihood ratio of a given fitting procedure, between 0 and 1) that reflects the result of this comparison and the goodness of fit: the greater the *p*DEV, the better the fit. A *p*DEV under 0.05 means an unacceptable fitting (for more details on this procedure, see Prins & Kingdom, 2018).

Separate analyses were performed to first compare conditions where the distance between the rubber hands and the actual hand is not equivalent (near-near vs. far-near vs. near-far) and the condition with equivalent distance (near-near vs. far-far). When necessary, the data were corrected to satisfy the sphericity assumption (Huynh–Feldt correction). One-factor (distance) repeated-measure analyses of variance (ANOVAs) were performed on the psychometric parameters, PSE and *σ*, with Holm’s post hoc tests for the significant effects. According to the central tendency theory (Ghasemi & Zahediasl, 2012), the number of observations in our paradigm (24 participants × 7 asynchronies × 3 or 2 distance conditions) was large enough to compensate for the small deviations from normality that could be observed in our data.

Finally, we used the light detectors mounted on the tips of the robots to consider any intrinsic delays in our system that could possibly have influenced our analysis. Specifically, the data from the light detectors gave us access to the actual estimated asynchrony between the touches seen on the rubber hands and the ones felt on the participants’ real hand. For each participant and each condition, the difference between the registered asynchrony and the theoretical (programmed) asynchrony was computed for the seven asynchronies (mean ± *SD*: 11 ± 8 ms). Given the small variability relative to the asynchronies used in the paradigm (50, 100, and 200 ms), we were satisfied with the precision of our robots and decided to use the theoretical (programmed) asynchronies in our analyses.

#### Control experiments (Fig. 2)

We reasoned that a putative concern with our paradigm could be that the participants might use some sort of strategy to solve the discrimination task instead of basing their decision on the perception of which hand felt most like their own. One possibility could be that they would choose the hand that is touched first (i.e., make their decision based on a visual judgment of the stimulation onset). Another possibility is that they could simply choose the hand they perceived as being touched at the same time as their real hand (i.e., they would perform a synchrony detection task). Of course, these strategies are unlikely given that the participants would then have to ignore our direct instructions, but we still wanted to make sure the participants were basing their perceptual decisions on body ownership and not some low-level sensory aspect of the timing of the stimuli.

**Fig. 2:**
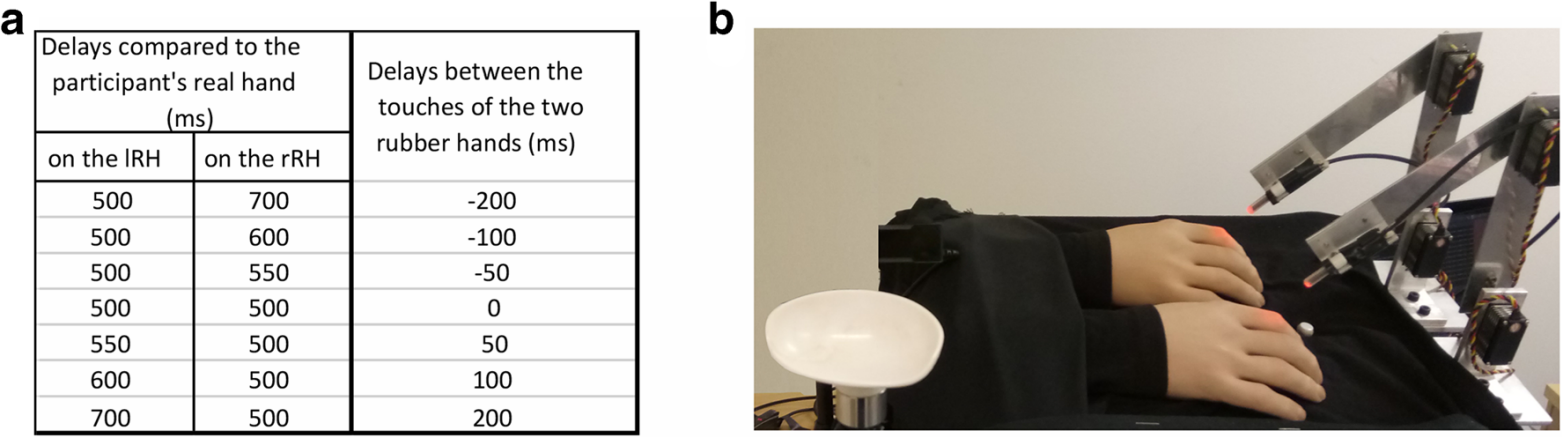
Experimental setup for the *wider asynchrony* condition (**a**) and the *rotated* condition (**b**). In the control experiment, the behavior in the *illusion* condition was compared with the behavior in two well-matched control conditions that did not elicit illusory ownership sensations: visuotactile stimuli with far too wide asynchronies (longer than 500 ms) (**a**) or with both rubber hands presented perpendicular to the participant real hand in anatomical implausible positions (**b**)

To control for this concern, we designed two control conditions with the same setup, but in which participants did not experience the RHI for either of the two rubber hands. Without illusory ownership, the question “Which hand feels the most like yours?” does not make sense, and the participants should therefore choose randomly between the rubber hands (i.e., independently of the asynchronies in the stimulation) if they followed the instruction. In contrast, if they followed an alternative strategy based on the timing of the stimuli as described above, their behavioral pattern should be the same in this control experiment as during the experiment with the RHI.

Therefore, this control experiment included three conditions: the *illusion* condition, the *wider asynchrony* condition, and the *rotated* condition. The *illusion* condition is identical to the near-near condition in the main experiment. In the *wider asynchrony* control condition, the only difference from the illusion condition is that the participants’ real hand was touched with a minimum asynchrony of 500 ms before any of the rubber hands where touched (see Fig. 2a); the degree of asynchrony was then systematically varied on the two rubber hands in seven steps up until a maximal value of 700 ms (500 ms, 550 ms, 600 ms, and 700 ms). Thus, compared with the *illusion* condition, the participants see exactly the same thing, but the tactile stimulation on the rubber hands is delayed to such an extent that the RHI should not arise for any of the tested asynchronies (Shimada et al., 2009). This control condition therefore addresses the question “Are the participants only detecting which rubber hand is touched first instead of reporting on their ownership percept?”.

The second control condition, the *rotated* condition, is the same as the *illusion* condition, except that the rubber hands are now rotated clockwise 90 degrees and placed perpendicular to the real hand. Thus, both rubber hands are placed in an anatomically incongruent position that prohibits the elicitation of the RHI (Costantini & Haggard, 2007; Ehrsson et al., 2004; Ide, 2013; Tsakiris & Haggard, 2005). Thus, the participants received the exact same visuotactile stimulation in the *rotated* condition as in the *illusion* condition (asynchronies varying from 0 ms to 200 ms), but they should not experience the RHI in the former condition. This control condition therefore answers the question “Are the participants only detecting which rubber hand is touched synchronously with their real hand instead of reporting on their ownership percept?”.

### Results of Experiment 1

#### Psychometric fitting based on temporal manipulation

**Fig. 3:**
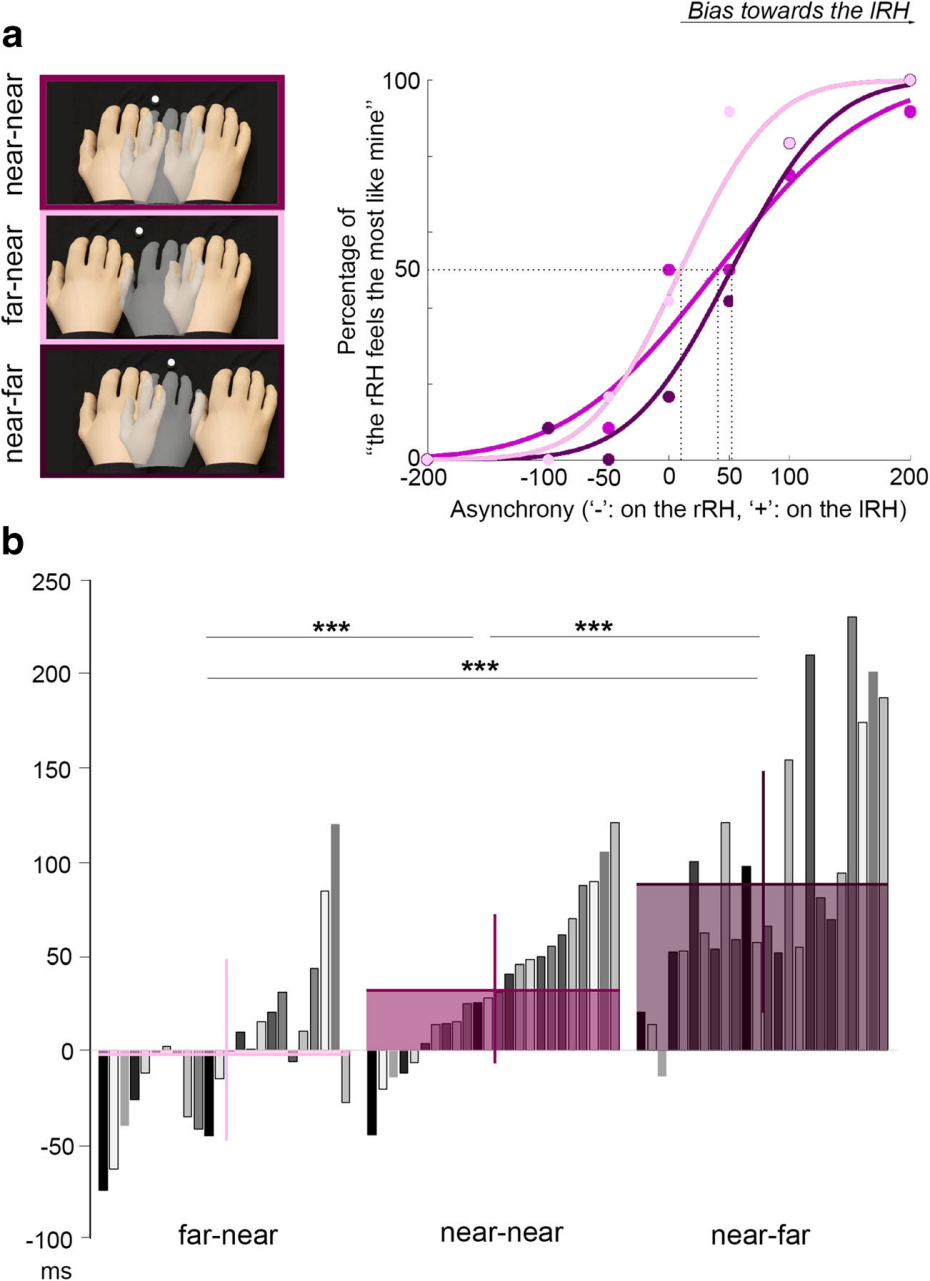
Changes in the psychometric curves across different conditions in Experiment 1. **a** Data collected for one representative participant (S14) in the near-near condition (purple curve), the far-near (light purple curve), and the near-far condition (dark purple curve). The changes in the PSE (i.e., the mean of the curves) reflect a bias in body ownership towards the rubber hand that is closer to the participant’s real hand. **b** Individual (gray bars) and mean (+*SD*, colored bars) extracted PSEs in the near-near condition (purple plot), the far-near (light purple plot), and the near-far condition (dark purple plot). A reduced PSE means a bias in body ownership in favor of the right rubber hand (rRH), while an increased PSE means a bias towards the left rubber hand (lRH). *p < .05. **p < .01. ***p < .001. (Color figure online)

In review, illusory ownership of the two rubber hands was elicited by a 12-s visuotactile stimulation period, during which one rubber hand was touched synchronously with the real hand while the other received asynchronous stimulation, where we systematically varied the degree of asynchrony in a stepwise manner up to 200 ms. The participants were then required to choose which of the two rubber hands (lRH or rRH) felt most like their own. The probability of choosing the rRH was successfully fitted by a cumulative Gaussian function for the tested asynchronies to obtain four psychometric curves per participant, one for each of the four conditions in Experiment 1 (see Fig. 3a for an individual example and Fig. S3 in the Supplementary Materials for all individual plots). Indeed, the goodness of fit for the obtained psychometric curves was assessed for each participant via the *p*DEV criterion. This criterion was above .1 for every participant, and the mean *p*DEV was .52 ± 0.3 (*p*DEV ± *SD* for the near-near condition: 0.54 ± 0.3, the far-near condition: 0.46 ± 0.2 near-far condition: 0.51 ± 0.2). Thus, the model chosen to fit our data is considered to be very satisfactory. Thus, it appears that the fine-grained manipulation of visuotactile asynchrony employed in the present study leads to systematic and specific changes in the ownership perception of the rubber hand in question, which is in line with the multisensory hypothesis of body ownership as outlined in the introduction. No significant effects of the participants’ sex on the fitting parameters were observed.

#### Spatial manipulation: Moving one rubber hand further away (Fig. 3)

In the near-near condition, both rubber hands are at the same distance from the real hand (5 cm in the horizontal plane), while in the far-near and the near-far conditions, respectively, either the lRH or the rRH was moved 5 cm further away. In line with our hypothesis, this distance manipulation significantly affected the observed PSE—that is, the mean of the psychometric curves, *F*(2, 46) = 55.7, *p* < .001, η^2^ = 0.71. Holm’s post hoc tests showed that compared with the near-near condition (34.7 ± 40 ms), the PSE decreases when the lRH is moved away (−0.5 ± 48 ms; near-near vs. far-near: Holm’s post hoc: *p* = .0003, *d* = 0.81) and increases when the rRH is moved away (93.8 ± 65 ms; near-near vs. near-far: Holm’s post hoc: *p* < .001, *d* = 0.96). This outcome means that when one rubber hand is moved away from a participant’s real hand, he or she will perceive equal ownership of both rubber hands when the touches on the rubber hand that is closer to the real hand are delayed by a moderate degree of asynchrony. In other words, the participants will favor the closer rubber hand over the one that is further away when making the ownership decisions. This spatial distance effect in body ownership perception is clearly shown by the shift in the psychometric curves’ PSEs. Another way to present these results is to calculate an “exchange rate” between the spatial and temporal asynchronies by dividing the change in the PSE between different condition by the change in the position of the rubber hands. Moving the lRH 1 cm away from the real hand causes a PSE reduction of 7 ± 6 ms (mean ± *SD*), and moving the rRH 1 cm away from the real hand causes a PSE increase of 12 ± 8 ms (mean ± *SD*), *t*(23) = 1.98, *p* = .064.

Notably, the PSE in the near-near condition is significantly greater than zero (one-sample *t* test), *t*(23) = 4.11, *p* < .001. This result means that when both rubber hands are touched synchronously with the real hand, the participant will, on average, prefer the lRH—that is, the one closer to the midline of the body. More precisely, 19 of 24 participants displayed this “midline bias” (see Discussion of Experiment 1 below; i.e., a positive PSE).

We now consider *σ* (i.e., the variance of the psychometric curves), reflecting the participants’ ability to choose between the two rubber hands in terms of which feels more like their own. The participants needed approximately 200 ms of asynchrony between the two rubber hands to show a clear preference for one of the model hands (near-near: 210 ± 90.4 ms; far-near: 207.1 ± 95 ms; near-far: 192.3 ± 98 ms). The manipulation of distance between the rubber hands did not significantly affect this minimum asynchrony, which is needed for the participants to clearly perceive a difference in ownership towards the two rubber hands, *F*(2, 46) = 0.48, *p* = .62, η^2^ = 0.02. Finally, we should emphasize that the results concerning the *σ*s also show a fine relationship between the timing of the visual and tactile events and body ownership, thereby confirming that body ownership perception also follows the temporal integration principle. Thus, the sensitivity of our test allows us to characterize both the spatial and temporal principles of multisensory integration in the RHI in a finer way than was possible in the previous literature (for further discussion, see Discussion of Experiment 1).

#### Spatial manipulation: Moving both rubber hands away (Fig. 4)

In the far-far condition, the lRH and the rRH are placed 10 cm to the left and the right of the participants’ real hand, respectively. Thus, the distances are matched in the near-near condition, but are 5 cm greater in each direction.

**Fig. 4:**
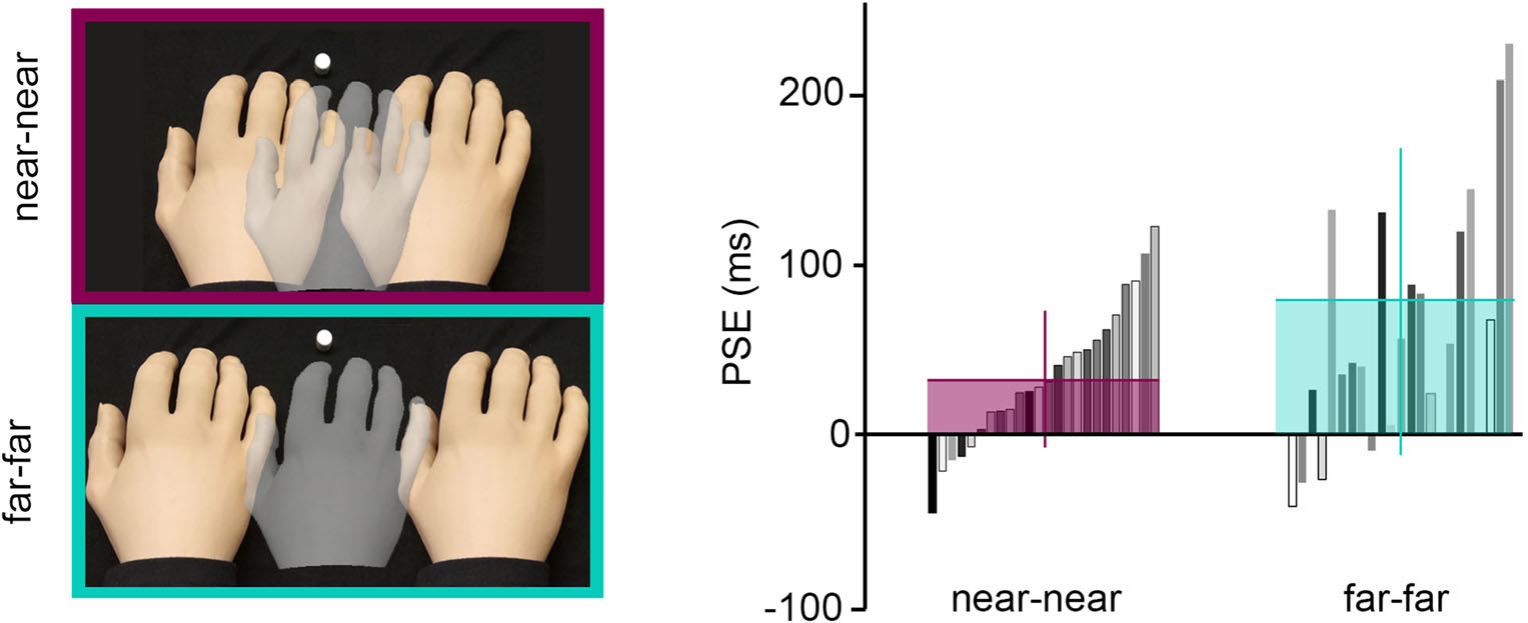
Changes in the psychometric curves across the near-near and far-far conditions in Experiment 1. Individual (gray bars) and mean (+*SD*, colored bars) extracted PSEs in the near-near condition (purple plot) and the far-far condition (cyan plot). A lower PSE indicates a bias in body ownership in favor of the right rubber hand (rRH), while a higher PSE indicates a bias towards the left rubber hand (lRH). Note that two of the 24 participants reported that they always felt that the lRH was theirs in the far-far condition (S2 and S13). The responses from these two individuals in this condition could therefore not be fitted by a psychometric curve, and the corresponding PSEs are therefore not plotted in the far-far histograms. *p < .05. **p < .01. ***p < .001. (Color figure online)

Two of our 24 participants reported that they always felt that the lRH was theirs in the far-far condition. This could either mean that they used a fixed response strategy, which is unlikely given that they did not use such a strategy in any of the other conditions, or that their bias or preference towards the most medially placed rubber hand (see Discussion of Experiment 1, below) was so strong that they always experienced the strongest ownership for this hand regardless of the asynchrony manipulation. Regardless of the underlying cause, these uniform responses made psychometric curve fitting impossible in these two individuals. Therefore, the comparison between the PSEs and *σ* of the curves was performed on the 22 remaining participants. In this pool of participants, while the mean PSE in the near-near condition was 27.4 ± 36 ms, it significantly increased up to 81.4 ± 95 ms in the far-far condition, *F*(1, 21) = 12.18, *p* = .002, η^2^ = 0.37. This result means that the bias in body ownership towards the rubber hand closer to the body midline is further increased when both rubber hands are further away from the participants’ real hand, causing the medial rubber hand (lRH) to be placed closer to the body midline and the lateral rubber hand (rRH) further away from the midline.

There were no significant differences in *σ* between the near-near and far-far conditions (near-near: 207.1 ± 96 ms; far-far: 311 ± 283 ms), *F*(1, 21) = 3.96, *p* = .06, η^2^ = 0.16. Thus, this type of distance manipulation did not significantly affect the minimum asynchrony needed for the participants to clearly perceive a difference in their ownership towards the two rubber hands.

We also control for time effect on the participants’ responses: the rRH/lRH answers ratio during the first half of one experimental block was compared with the rRH/lRH answers ratio during the second half of the same experimental block. No significant difference was found, regardless of the condition (Wilcoxon paired test: near-near: V = 74, *p* = .15; far-near: V = 129, *p* = .95; far-far: V = 52, *p* = .26; near-far: V = 101, *p* = .90).

#### Control experiment

To further ensure that our participants truly based their behavioral responses on the perceived ownership of the hands and not an alternative strategy based on the timing of the stimuli, we compared the data acquired in the *illusion* condition (identical to the near-near condition presented above) and two control conditions displaying the same timing relationship between the seen and felt stimulations, but where RHI should not arise—the *wider asynchrony* condition, with a range of delays between visual and tactile stimuli that were all too long to elicit the illusion, and the *rotated* condition, where the model hands are placed in anatomically implausible positions (see Control Experiment section, above, for details).

The responses obtained in the *illusion* condition were well fitted by a cumulative Gaussian function, just as the near-near condition in the main experiment described above (mean PSE ± *SD* = −15 ± 61 ms, *p*DEV ± *SD* = 0.89 ± 0.1). At the behavioral level, this confirms the conclusion from Experiment 1 that the participants’ perception of ownership of the rubber hands under different degrees of asynchrony within the 0 ms to 200 ms time window can adequately be described by a cumulative Gaussian distribution.

**Fig. 5:**
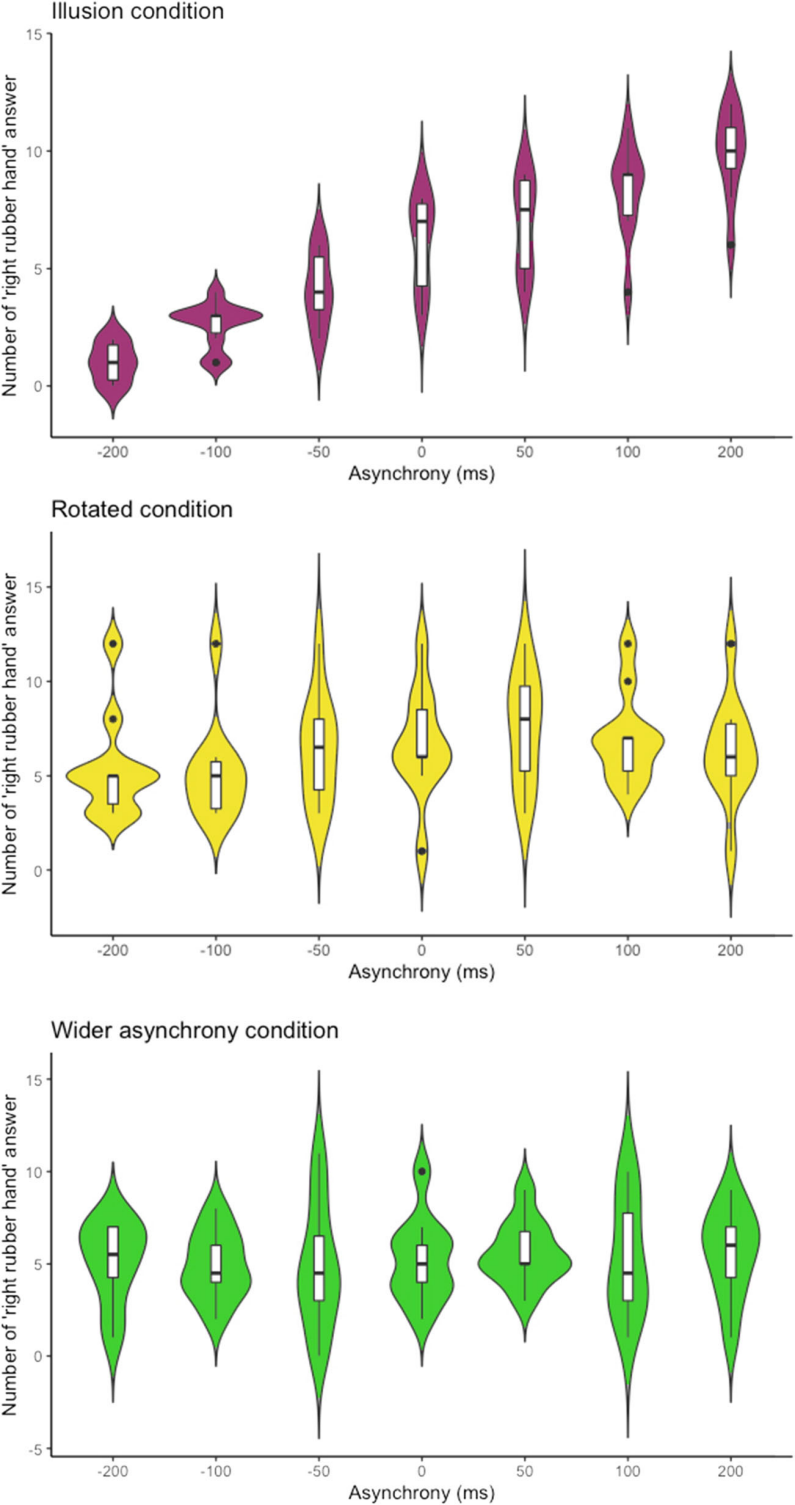
Results from the control experiments. The plots represent the number of times the participants choose the right rubber hand under different conditions of asynchrony: illusion (purple), rotated (yellow), and wider asynchrony (green) conditions. These results are shown for purely descriptive purposes; the assessment of RHI was based on the fit (or lack of fit) of each participant’s data to the Gaussian cumulative function as described in the main text. The plotted colorful shapes reflect the probability density of the corresponding answer among the participants. The lower and upper hinges of each box inside the shapes correspond to the first and third quartiles, respectively, with the thick horizontal lines representing the medians. The upper and lower whiskers extending from the boxes indicate the range of maximum to minimum values. A post hoc test revealed a significant relationship between the level of asynchrony and the frequency of right rubber hand responses only in the illusion condition (*illusion* condition: *χ*^2^ = 170.06, *df* = 6, *p* < .001; *rotated* condition: *χ*^2^ = 11.96, *df* = 6, *p* = .06; *wider asynchrony* condition: *χ*^2^ = 1.85, *df* = 6, *p* = .93). (Color figure online)

In contrast, and as hypothesized, in the *wider asynchrony* and the *rotated* conditions, the model loses its behavioral relevance as no ownership experiences were elicited (see Fig. 5). If we force the fitting for those data, we obtain mean PSEs of 4,157 ms (*SD* ± 12 987) and 188,232 ms (*SD* ± 550 778) for the *wider asynchrony* and the *rotated* condition, respectively. In addition to an unsettling degree of interindividual variability associated with this fit, these values make no sense and are much higher than would be expected if ownership were involved in the decisions. These results simply show there is no meaningful pattern in the participants’ responses. Moreover, these values are much too high to correspond to discrimination values expected in a visuotactile synchrony detection task (Shimada et al., 2014). Thus, our participants did not use a synchrony detection strategy to decide which of the two rubber hands felt more like their own. Moreover, the mean goodness of fit of our analyses drops from 0.89 ± 0.1 in the *illusion* condition to 0.42 ± 0.2 and 0.39 ± 0.3 in the *wider asynchrony* and the *rotated* conditions, respectively, even if the fitting parameters such as the PSE are free to take values outside of what is behaviorally relevant. Therefore, in the control conditions, the psychometric fitting loses its goodness of fit as well as its behavioral significance in comparison to the *illusion* condition. This loss of psychophysical model fitting is further illustrated by calculating the Bayesian information criterion (BIC) for each condition in the control experiment. The lower the BIC, the better the fit. Importantly, we observed significantly lower (*p* < .05) BIC in the *illusion* condition compared with the *wider asynchrony* or *rotated* conditions (see Table 1) and this further validates that the model fit was best with the data from the illusion condition. Altogether, the above results confirm that the psychometric fitting of our participants’ responses regarding body ownership is relevant only when the RHI is induced and that our participants were not using an alternative strategy based on the timing of the stimuli when reporting their decisions about ownership perception.

**Table 1:** Bayesian information criterion (BIC) used to statistically compare the cumulative Gaussian fit in the different condition of the control experiment

**Paired** ***t*** **test**	*Wider asynchrony* (BIC = 145 ± 49)	*Rotated* (BIC = 141 ± 25)
*Illusion* (BIC = 92 ± 10)	*t*(9) = 2.87, *p* = .023	*t*(9) = 5.13, *p* = .001

### Discussion of Experiment 1

In the first experiment of the present study, participants performed a discrimination task on the ownership they felt towards two identical right rubber hands; the tactile stimulation they felt on their hidden real hand was synchronized with one of the rubber hands, the other rubber hand receiving a stimulation systematically delayed in steps from 0 ms up to 200 ms, or both rubber hands received synchronized stimulation. For each degree of asynchrony, the percentage of trials for which the participants choose the rRH as theirs was collected. These collected data were well fitted by a cumulative Gaussian. Participants’ perception of body ownership can therefore be quantitatively described by two parameters: the mean (i.e., the PSE) and the variance (*σ*) of the psychometric curves computed from the participants’ answers. This fitting did not work if we used very long asynchronies between the visual and the tactile stimulation (>500 ms) or if we rotated the rubber hands by 90°, as evidenced in the control experiment. These latter manipulations eliminate the RHI, and thus these latter results exclude the possibility that the participants were basing their perceptual decisions on the temporal properties of the visual and tactile signals rather than on the ownership percepts. Thus, collectively, the results from Experiment 1 demonstrate that body ownership can be assessed reliably as perception in a two-alternative forced-choice discrimination task.

With this new paradigm, we were also able to examine the spatial and temporal congruency constraints of the RHI with a finer resolution than have been reported with the traditional methods of questionnaires and proprioceptive drift. In the spatial domain, we found that placing one rubber hand just 5 cm further away from the participant’s real hand in the horizontal plane decreases the illusion for this model hand, which was in line with our hypothesis and the multisensory integration account of body ownership. In our paradigm, this effect was shown by a shift in the psychometric curve mean (PSE)—that is, the RHI remains stronger for the rubber hand that is closer to the hidden real hand despite larger asynchronies between what is felt and what is seen. Notably, this diminishing effect on the RHI was observed for a distance that was substantially smaller than the value of approximately 30 cm identified as the distance that broke the illusion in earlier studies using traditional measures (Kalckert & Ehrsson, 2014; Lloyd, 2007; Preston, 2013). Moreover, in these previous studies, the position of the rubber hand was varied in steps of a minimum of 10 cm. To our knowledge, this experiment is the first to assess the size of this effect directly and quantitatively for such small variation in the rubber hand position (5 cm). Our result fits well with the definition of body ownership as a perceptual phenomenon that emerges from multisensory integration: the strength of the RHI is affected by the degree of conflict between the sensory inputs, including the proprioceptive inputs from the real hand and the visual feedback from the rubber hand, each conveying different information about the hand position (see the General Discussion section below).

Our results also demonstrate the high sensitivity of our paradigm in the temporal domain, shedding new light on the link between visuotactile asynchrony and the RHI. Indeed, in their 2009 study, Shimada et al. showed that only delays of more than 300 ms significantly reduced the RHI. Thus, with the current novel approach, we are able to go beyond the observations made by Shimada and demonstrate that visuotactile asynchronies as short as 200 ms are sufficient to significantly affect the ownership that our participants feel towards the rubber hands, as shown by the discrimination threshold (*σ*) results. Thus, the RHI seems to have a narrower temporal window of integration than previously thought. Relative to previous results, our new results better fit the temporal congruency principle of multisensory integration in that they are more similar to the asynchronies detected in visuotactile simultaneity tasks (Costantini et al., 2016; Noel, Wallace, Orchard-Mills, Alais, & Van der Burg, 2015; Vroomen & Keetels, 2010) and the neuronal temporal window of integration of visuotactile signals in multisensory cortex (in the posterior parietal lobe; Avillac, Ben Hamed, & Duhamel, 2007).

Finally, we noted a spatial bias in our result: When the two rubber hands were positioned at the same distance from the real hand, the lRH, which was closer to the participant’s torso and body midline, was favored compared with the rRH, which is placed farther from the body midline, as shown by the majority of positive PSEs in the near-near conditions and the far-far conditions. Such a spatial bias for rubber hands placed closer to the torso and body midline has been observed in the previous literature on body ownership (Fan & Ehrsson, 2020; Newport et al., 2010; Preston & Newport, 2011), although the underlying cause is not fully understood. One recent study found that multisensory integration in the context of the RHI decreases when the distance to the body increases even if the distance between the real hand and the visible hand stays identical (Dempsey-Jones & Kritikos, 2017). Furthermore, we know that visual and tactile signals are more likely to be integrated when occurring close to the body midline than further away (Mirams, Poliakoff, & Lloyd, 2017; Van der Biest, Legrain, Paepe, & Crombez, 2016), which should have an impact on body ownership (Makin et al., 2008). In light of these previous findings, it might be no surprise that in our experiment the rubber hand presented closer to the body midline was associated with a positive spatial bias compared with the one placed further away, although the distance to the real right hand was identical. (In the near-near and far-far conditions, the lRH is always within the peri-personal space from the midline of the trunk [25 and 20 cm, respectively], while the rRH reaches the limit of the peri-personal space of the trunk [35 and 40 cm away from the body midline, respectively]. Importantly, the midline bias effect was greater in the far-far condition than in the near-near condition, which could be related to the rRH being at the boundary of the peri-hand space in the former condition in addition to being further from the body midline.) However, it is important to emphasize that the bias towards the medially placed rubber hand does not affect any of our main findings with regard to the temporal and spatial congruency effects in Experiment 1. Although there was a midline bias in the PSEs of our participants in the near-near condition, we still observed a shift of these PSEs in the far-near and near-far conditions, meaning that the preference for the lRH was reduced when the rRH was closer to the real hand and increased when the lRH was closer to the real hand.

In summary, based on the results from Experiment 1 and the associated control experiment, we feel confident that our psychophysical discrimination task characterizes body ownership as genuine perception in the RHI paradigm and that our results thus shed light on core perceptual mechanisms involved in body ownership. To further exploit the possible superior sensitivity if this method, we next investigated the influence of tactile incongruence between the seen and felt touches, testing the hypothesis that even relatively small incongruencies of this type should impact the RHI (see the Introduction).

## Experiment 2

In the second experiment, we used our newly developed psychophysical paradigm to assess how congruence between the material of the objects used to touch the rubber hands and the real hand influence the sense of body ownership. As described in the Introduction, this type of experimental manipulation has led to contradictory findings in the literature (Schütz-Bosbach et al., 2009; Ward et al., 2015), which could relate to the inherent limitations of the traditional methods used to measure the RHI (see the Introduction). In line with the multisensory hypothesis of body ownership, we predicted that the tactile congruency between the seen and felt touches should be a basic factor that influences ownership percepts in the RHI paradigm.

### Method

#### Participants

Thirty healthy participants were recruited for the second experiment (15 females, ages 24.8 ± 5 years). Five of the subjects had participated in Experiment 1.

#### Inclusion test

The participants underwent the same pretesting session as in Experiment 1 to confirm that they could experience a basic RHI using the same criteria as described above. Eight participants (two females) did not reach the inclusion criterion; therefore, 22 participants were included in the study (for details, see Table S2 in Supplementary Material).

#### Experimental setup

In Experiment 1, the participant was required to position his or her right hand 30 cm away from the body midline. This position was a comfortable configuration of the right arm for most participants, but not a completely ecological resting position for the shortest volunteers. Thus, to make the psychophysical procedure as comfortable as possible for all participants and allow them to adopt an ideally relaxed posture of the right arm, we changed two elements of the original setup. First, we brought the whole setup closer to the participants’ body midline. Thus, their hand was now 22 cm away from their body midline. This change made it easier for the participants to relax their right shoulder throughout the relatively long experimental sessions. Second, we tilted the whole setup with the rubber hands, table, robots, and the participants’ real hand 30° upwards in the sagittal plane (from the horizontal plane). This change made it easier than before for the participants to view the rubber hands, facilitating the relaxation of the arm and shoulder and further aiding the participants in maintaining their gaze at the fixation point. The rest of the setup was identical to that of Experiment 1.

#### Procedure

The procedure was identical to that of Experiment 1, with the only difference being that we manipulated the tactile congruence of the objects touching the real and rubber hands rather than the distance between the model hands. Ownership of the two rubber hands was elicited by 12-s visuotactile stimulation periods, where both rubber hands were touched synchronously with the participants’ real hand in the same sequence of six locations on the index finger as used in Experiment 1 (see Procedure section in Experiment 1), or one of the rubber hands was touched with a small degree of asynchrony that varied in four steps up to 200 ms, as in Experiment 1. However, in contrast to Experiment 1, the distance between the rubber hands and the participant’s real hand was not varied but remained the same across conditions (5 cm). Instead, we manipulated the tactile congruence between the objects touching the rubber hands and the object used to touch the participant’s hidden real hand. To this end, two different materials were used for the endings of the robot arms touching the hands: firm plastic (identical to Experiment 1) and polyethylene foam (see Fig. 4a, left panel). Both types of endings were the same size and shape—cylindrical with a 7 mm diameter—and were flexible enough to bend slightly when touching the rubber hands or the real hand. Thus, the contact surface between the endings and the skin was matched for plastic and foam endings, meaning that these stimuli differed only in terms of their material texture. Three tactile congruence conditions were tested: (a) “congruence” (the materials of the objects touching the real hand and both rubber hands are the same); (b) “left incongruence” (the materials of the objects touching the real hand and the rRH are the same, and the material of the objects touching the lRH and the real hand is different); (c) “right incongruence” (the materials of the objects touching the real hand and the lRH are the same, and the material of the objects touching the rRH and the real hand is different). Altogether, six conditions were tested, where we varied the object touching the real hand and the tactile congruence (2 materials × 3 *congruence* conditions), which meant six blocks of 27 minutes for each participant. As during Experiment 1, the order of the blocks was counterbalanced between the participants. The participants never saw which material touched their real hand. Within each block, the different delays were present in random order, each repeated 12 times. Thus, in each block, participants made 84 judgments of ownership between the rRH and the lRH. The probability of choosing the rRH was fitted by a cumulative Gaussian function for the tested asynchronies to obtain six psychometric curves per participant.

#### Data analysis

To evaluate and compare participants’ perception across the six conditions (2 materials × 3 *congruence* conditions), the psychometric data (i.e., the proportion of “the right hand feels the most like my hand” answers at different asynchronies) were fitted by a cumulative Gaussian function. The same fitting method used in Experiment 1 was employed to compute the psychometric curves for each participant in each condition (see Data Analysis section for Experiment 1). Upon reviewing the light-detector data, we found that the variability in the real onset of the different taps was small enough (mean ± *SD*: 12 ± 6 ms) for us to always use the programmed theoretical asynchronies in the fitting.

A two-way repeated-measures ANOVA was performed on the psychometric parameters, PSE and *σ*, with Holm’s post hoc tests to assess the impact of the materials and the *congruence* factors on the participants’ body ownership perception.

### Results

The participants’ probability of choosing the rRH as theirs was successfully fitted by a cumulative Gaussian function for the tested asynchronies to obtain six psychometric curves per participant (goodness of fit: mean *p*DEV ± *SD*: 0.45 ± 0.3). The fitting worked well in all participants, which confirms the basic finding from the first experiment that the influence of the degree of visuotactile asynchrony on the ownership decisions can be described well by a cumulative Gaussian distribution. No significant effects of the participants’ sex on the fitting parameters were observed.

**Fig. 6:**
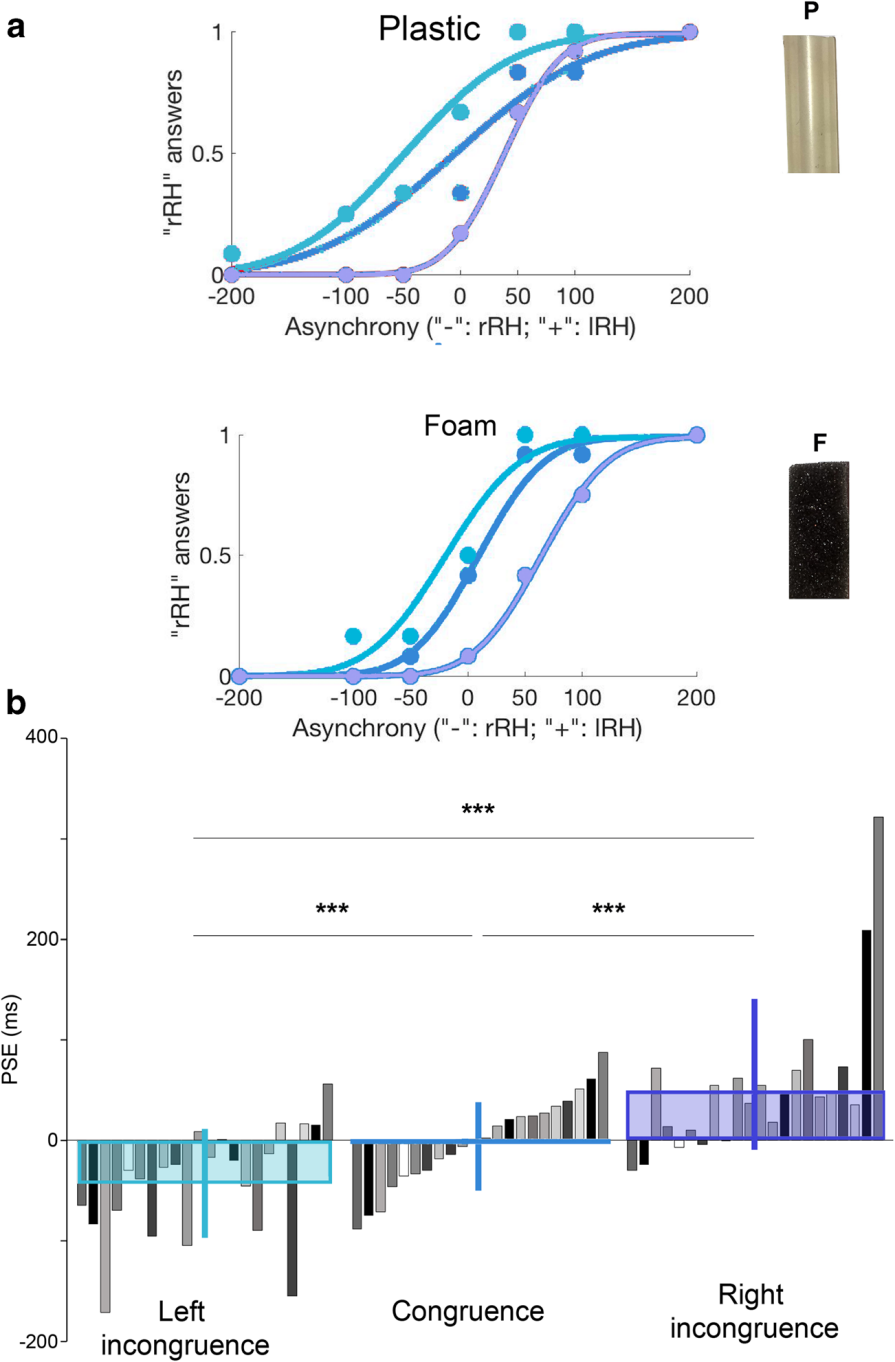
Changes in the means of the psychometric curves across the different congruence conditions. **a** The tips with which the robots touched the real and rubber hands were made from plastic or foam. Six different combinations of materials touching the real and rubber hands were tested in a 2 × 3 factorial design with three levels of congruence: the congruence condition (all hands touched with the same material), the left incongruence condition (the left rubber hand [lRH] was touched with a different material), and the right incongruence condition (the right rubber hand [rRH] was touched with a different material). Data were collected for one representative participant (S14) in the left incongruence condition (turquoise curve), the congruence condition (blue curve), and the right incongruence condition (indigo curve) when the tip touching the real hand was plastic (upper curves) or foam (lower curves). The changes in the PSE—that is, the rightward or leftward shifts in the mean of the curves, reflect a bias in body ownership towards the rubber hand that is touched by the same material as the participant’s real hand. **b** Individual (gray bars) and mean (+*SD*, colored bars) extracted PSEs in the congruence condition (blue plot in the middle), the left incongruence condition (turquoise plot on the left), and the right incongruence condition (indigo plot on the right). A reduced PSE means a bias in body ownership in favor of the rRH, while an increased PSE means a bias towards the lRH. The observed pattern of shifts in PSE indicates that introducing tactile incongruence in stimulation of one rubber hand shifted ownership towards the other rubber hand, as we had predicted. *p < .05. **p < .01. ***p < .001. (Color figure online)

The observed behavior was not significantly different depending on whether the touches applied to the real hand were delivered by plastic or foam tips (i.e., there was no main effect of object material in our factorial design), *F*(1, 21) = 0.85, *p* = .37, η^2^ = .04. As hypothesized, however, introducing an incongruence in the stimulation of one rubber hand biased the participants’ perception towards the other rubber hand, *F*(2, 42) = 26.26, *p* < .001, η^2^ = .56 (see Fig. 6b). Indeed, the PSE in the left incongruence condition (mean ± *SD*: −42.5 ± 62 ms) was significantly smaller than the PSE in the congruence condition (mean ± *SD*: −1.4 ± 51 ms; Holm’s post hoc: *p* = .003, *d* = 0.75), which, in turn, was also smaller than the PSE in the right incongruence condition (mean ± *SD*: 54.6 ± 96 ms, Holm’s post hoc: *p* < .001, *d* = 0.82). There was no significant interaction between the two experimental factors, *F*(2, 42) = 0.08, *p* = .87, η^2^ = .004, meaning that it was the tactile incongruency *per se* that was driving the above effects, irrespective of material.

In accordance with our hypothesis and the results from Experiment 1, the experimental manipulations did not significantly impact the *σ*s: material, *F*(1, 21) = 0.22, *p* = .88, η^2^ = .001; congruence, *F*(2, 42) = 1.19, *p* = .31, η^2^ = .054; Material × Congruence, *F*(2, 42) = 1.32, *p* = .28, η^2^ = .059. Thus, neither the material nor the tactile congruence of the objects influenced the minimum asynchrony needed for the participants to clearly perceive a difference in their ownership towards the two rubber hands. On average, a minimum of 170 (*SD* ± 80) ms of asynchrony was sufficient for our participants to perceive a clear difference in ownership between the left and the right rubber hands.

Notably, we did not observe a bias towards the body midline in the congruence conditions. The PSEs in these conditions did not differ significantly from zero (one-sample *t* test against 0), plastic congruence: *t*(21) = .071, *p* = .49; foam congruence: *t*(21) = .026, *p* = .75. An a posteriori analysis showed that the mean PSE for the congruent conditions in Experiment 2 was significantly smaller than that in the near-near condition Experiment 1 (two-sample *t* test), *t*(44) = 141, *p* = .007, *d* = 0.78. We speculate that this difference in “midline bias” across the two experiments relates to the different spatial arrangements of the hands used in the two versions of our setup (see Discussion of Experiment 2, below). Figure 6.

### Discussion of Experiment 2

Experiment 2 used the same basic paradigm as Experiment 1, but manipulated the tactile congruence between the objects touching the rubber hands and the participant’s real hand. Before turning to these main novel results, we first note that, as in Experiment 1, the psychometric curves obtained from the participants’ responses were the result of a robust and satisfying fitting that confirms the effectiveness of our behavioral method in quantifying body ownership. Once again, the judgment of the participants regarding their ownership of the rubber hands was influenced by asynchronies smaller than the 300-ms time window reported in earlier studies (Shimada et al., 2009). According to our *σ* results, the participants in this study needed a minimum of 170 ms of visuotactile asynchrony to discriminate ownership efficiently between the two rubber hands, which is similar to the approximately 200-ms discrimination threshold observed in Experiment 1.

When one of the rubber hands was touched with a probe having the same size and shape but made from a different material than the ones touching the real hand and the other rubber hand, the corresponding psychometric curve mean (PSE) significantly differed from the conditions when all robot material endings were identical. Thus, even small discrepancies in tactile congruency between the seen and felt objects touching the hands lead to a significant reduction in body ownership. Earlier experiments employing questionnaires and proprioceptive drift have failed to detect such a tactile incongruence effect (Schütz-Bosbach et al., 2009) or have found such an effect only for very great incongruencies where the objects touching the hands differed along multiple macrogeometric (shape, size) and microgeometric (texture) perceptual dimensions (Ward et al., 2015). In the study by Ward et al. (2015), when two objects that seemed to differ only in texture (roughness and hardness) were used—a soft versus a hard brush—no tactile congruence effect was found in the questionnaire ratings or the proprioceptive drift. In line with this uncertainty, a very recent study (Filippetti, Kirsch, Crucianelli, & Fotopoulou, 2019) found a significant tactile affective congruence effect when comparing Velcro fabric and synthetic wool on an embodiment questionnaire, but found no such differences in proprioceptive drift, although the two fabrics tested also differed in texture and experienced pleasantness. In addition to these mixed results, this latter study differs from the present and the aforementioned ones in that the tactile stimulation was purposefully delivered within the optimal speed range of the CT fibers (i.e., slow, pleasant stroking). The findings of Filippetti et al.’s (2019) study may thus relate both to the potential involvement of affective touch in body ownership and a potential effect of texture congruence. In the present study, the tactile stimuli (taps) were not designed to activate CT fibers or create affective experiences. Therefore, our study isolates perceptual congruence effects by avoiding the elicitation of any potential affective responses. Our results show that even a small and purely tactile incongruence between two objects that differ only in terms of texture influences the RHI. This finding suggests that our psychophysical approach is more appropriate than previous methods used to study the influence of the tactile congruency of the objects touching the hands in the RHI paradigm. Conceptually, this finding is important because it suggests that body ownership follows the same principles as other types of multisensory perception, for which the strength of the percept depends not only on temporal and spatial congruency but also on multisensory congruency along other dimensions of the stimuli.

Unlike in the Experiment 1 results, we observed no significant preference for the more medial of the two rubber hands (lRH) when the stimulation was congruent. We speculate that this difference across the two experiments could be due to the changes we made in the placement of the real and rubber hands in the second experiment. We interpreted the bias towards the more medially positioned rubber hand (lRH) in Experiment 1 as a consequence of the rRH reaching the limits of the peri-body space and thus becoming less likely to be chosen as one’s hand (as shown by the positive mean PSE in the near-near condition; Brozzoli et al., 2014; Guterstam et al., 2013). In Experiment 2, the real hand and both rubber hands were closer to the body midline than in Experiment 1 (<25 cm instead of <35 cm), and the whole setup was tilted 30 degrees. Thus, in this version of the paradigm, both rubber hands are fully included in the peri-body space, and no systematic preference for the most medial rubber hand is observed. The reduction in the perceptual bias in Experiment 2 is therefore consistent with our interpretation of the midline bias finding of Experiment 1 as discussed above (see Discussion of Experiment 1 section). Nevertheless, the present study was not designed to address this specific question, and future experiments could reexamine this issue by systematically manipulating the locations of the rubber and real hands with respect to peri-body space centered on the trunk, the hand, the shoulder, or the head.

## General discussion and conclusion

This study presents a new methodological approach to register the sense of body ownership via a 2-AFC discrimination task that involves the RHI with two concurrently stimulated rubber hands. Our results suggest that this method produces direct, sensitive, and robust measurements of hand ownership, thus providing an alternative approach to investigate body ownership that has several advantages with respect to traditional methods. Crucially, 2-AFC discriminations of body ownership should be less susceptible to cognitive bias than questionnaires and relate more directly to perception than the proprioceptive drift index and the threat-evoked SCR. A further important point is that our findings advance our conceptual understanding of the temporal, spatial, and tactile congruence principles that determine body ownership. The temporal and spatial incongruencies that can be tolerated without reducing the RHI are shorter and smaller than previously reported with traditional methods, and these new results confirm predictions from theoretical models of multisensory integration (Choi, Lee, & Lee, 2018; Colonius & Diederich, 2020; Stein, 2012). Similarly, important from this theoretical perspective, our results show that the tactile congruency between the seen and felt objects touching the two hands constitutes a basic perceptual constraint of the illusion, a finding that resolves a controversy in the previous literature (Schütz-Bosbach et al., 2009; Ward et al., 2015). Collectively, these results provide important empirical support for the hypothesis that body ownership can be explained as multisensory perception of one’s own body.

### Body ownership as multisensory perception assessable by psychophysics

As explained in the Introduction of this paper, the lack of rigorous behavioral paradigms to directly register body ownership has been a major obstacle to conceptual advances in the field (Longo et al., 2008). The present study contributes to solving this problem by demonstrating that body ownership can be studied as perception through a new psychophysical approach in which a participant must decide which of two simultaneously presented and stimulated rubber hands feels more like his or her own. This discrimination method addresses many of the concerns associated with the classical measures of body ownership, such as the lack of specificity of the proprioceptive drift and the SCR, and the many biases that can affect the questionnaire ratings (see the Introduction). Moreover, assessing ownership discrimination for two simultaneously presented identical body parts, instead of examining the mere presence or absence of the illusion for a single body part (detection), allows the determination of classic psychophysics variables such as the point of subjective equality (PSE) and just-noticeable difference (JND). Although questionnaires can certainly continue to be used as a quick and easy way to register the presence of the subjective RHI, the present psychophysics approach is more suitable to address questions that relate to the specific perceptual processes of body ownership and for quantitative model testing (see further below). The present method also allows to increase in the number of repetitions of the experimental condition compared with traditional techniques such as the use of questionnaires, leading to a robust measurement of the RHI (for a recent study of the importance of intraindividual variability in multisensory processing, see Murray, Thelen, Ionta, & Wallace, 2019). Collectively, the results of the two current experiments and the associated control experiments provide consistent evidence that our psychophysical discrimination task adequately and accurately registers body ownership as multisensory perception.

Perception refers to a complex and yet primary operation through which individuals organize their sensory inputs into a representation of the objects and the events that surrounds them (Efron, 1969), and body perception extends this definition to the bodily self. Psychophysics examines the direct relationship between the quantitative manipulation a physical stimulus and the corresponding induced perception in an observer (Kingdom & Prins, 2009). In the present experiments, the quantitative manipulation involved the temporal congruence of the visual and tactile stimuli, and the induced perception corresponded to the sensation of the rubber hands as being part of one’s own body (body ownership). In both Experiment 1 and Experiment 2, the participants’ forced ownership decisions under several conditions of visuotactile asynchrony were adequately described by psychometric curves. Importantly, the good fit of our behavioral data to the psychometric curves and the consistency of our finding to basic spatiotemporal constraints of body ownership indicates that body ownership can be discriminated in this way by the participants. Thus, our results suggest a direct and continuous link between the systematic manipulation of visuotactile synchrony (physical stimulus) and the resulting body ownership discriminations. This finding argues in favor of body ownership as perception, as opposed to cognition such as sematic categorization of “my body” versus “not my body” or judgements based on conceptual reasoning or by referring to abstract knowledge about one’s body.

Our findings and conclusions have important bearings on future computational modeling of body ownership. Bayesian models of multisensory integration have been efficiently used to describe the integration of inputs from different sensory modalities in many perceptive tasks, including audiovisual integration (Alais & Burr, 2004), visuohaptic integration (Ernst & Banks, 2002), and the integration of somatosensory and visual signals from the body (Chancel, Blanchard, Guerraz, Montagnini, & Kavounoudias, 2016; Reuschel, Drewing, Henriques, Rösler, & Fiehler, 2010; van Beers et al., 2002). However, few attempts have been made thus far to quantitatively apply this model to body ownership perception (Fang, Li, Qi, Li, Sigman, & Wang, 2019; for a qualitative approach, see Samad et al., 2015). These studies have pioneered our understanding of the computational principles of body ownership (Ehrsson & Chancel, 2019), although they relied on questionnaire ratings and proprioceptive drift as measures that limit the conclusiveness of their findings. For example, Fang et al. (2019) performed quantitative modeling of hand position errors in a pointing task (that resembles the proprioceptive drift task) and showed that a “causal inference model” could better explain the integration of visual and proprioceptive signals from the hand than a “forced fused model.” However, these findings could reflect the localization of the arm in space rather than be specifically related to the perception of hand ownership. Fang and colleagues also collected questionnaire data, but could only relate these to the causal inference model by a simple correlation between subjective rating of the illusion and the estimated model parameters based on the indirect proprioceptive drift measurements. The new quantitative modeling presented in the present article open up new avenues for directly testing quantitative computational models of body ownership that incorporate varying signal reliabilities and noise in different sensory channels, maximum likelihood estimation, and causal inference.

### Refining knowledge of the spatial and temporal constraints of body ownership

The two experiments presented here go beyond the previous studies on the temporal and spatial constraints of RHI, and we will first consider how our results nuance our understanding of the temporal window of visuotactile integration affecting the RHI (Shimada et al., 2009; Shimada et al., 2014). Indeed, in our two experiments, a visuotactile asynchrony of approximately 200 ms is sufficient to significantly change the ownership perception in our participants, while previous studies observed no significant diminishing effect on the RHI until the visuotactile asynchronies were larger than 300 ms (Shimada et al., 2009; Shimada et al., 2014). This difference in results probably relates to the increased sensitivity and precision of the current two-alternative forced-choice discrimination task. Alternatively, the longer stimulation periods used in the Shimada studies (60 to 180 s compared with 12 s in the current experiments) could have allowed for an illusion to slowly build up even under the greater asynchronies due to temporal correlation (Burr, Silva, Cicchini, Banks, & Morrone, 2009; Parise & Ernst, 2016; Parise, Spence, & Ernst, 2012). Regardless of the reason for this difference, the narrower temporal window for body ownership identified in the current study better fits the temporal congruency principle of multisensory integration because it is closer to the temporal windows of integration observed in behavioral tasks (Costantini et al., 2016; Noel et al., 2015; Vroomen & Keetels, 2010) and in neurons in multisensory cortex performing visuotactile integration (Avillac et al., 2007).

In Experiment 1, we also investigated the spatial constraints of the RHI. Importantly, we found a reduction in reported ownership when a rubber hand was placed 10 cm away from the real hand in the horizontal plane compared with 5 cm away. Such a spatial incongruency effect for a change in distance of only 5 cm between the seen rubber hand and the hidden real hand has not been detected in the earlier literature, where classical measurement methods were employed (Kalckert & Ehrsson, 2014; Kalckert et al., 2019; Lloyd, 2007; Motyka & Litwin, 2019; Preston, 2013; Zopf et al., 2010), and yet fits better with the high accuracy of individuals in perceiving the previously observed position of their arm (Paillard & Brouchon, 1968.; Walsh, Hesse, Morgan, & Proske, 2004). The more fine-grained spatial effect that could be revealed with the current psychophysics approach is more consistent with a multisensory account of body ownership because even small incongruencies between the seen and felt location of the hand should influence the integration of visual and somatic signals from the limb (van Beers, Sittig, & Gon, 1999; van Beers et al., 2002), and according to causal inference and optimal integration models of multisensory integration (Ehrsson & Chancel, 2019; Körding et al., 2007; Samad et al., 2015), any conflict between the sensory signals available to the participants, however minimal, should decrease the probability of them coming from the same source. As a result, the integration of these signals into a unified percept should be weakened even for subtle spatial incongruencies. Thus, if the RHI is the percept emerging from the integration of vision, touch and proprioception as we argue, an incongruence between these sensory signals should always result in a decrease in the corresponding ownership percept. Our results when manipulating the distance between the real hand and the rubber hands match this critical prediction. It is important to stress that the spatial congruence effect we observed occurred when both rubber hands were placed within peri-hand space. Earlier studies have demonstrated that when the rubber hand is presented outside peri-hand space—approximately 30 cm or further away from the real hand—the illusion is significantly diminished (Kalckert & Ehrsson, 2014; Lloyd, 2007). Thus, in addition to this peri-personal space constraint (see also Guterstam et al., 2016) that determines the furthest distance for which the illusion can be elicited under optimal conditions, the present results have revealed a more general spatial congruency principle that relates to basic degree of spatial (in)congruency between the visual, proprioceptive and tactile information regarding the hand *within* peri-personal space. The existence of such a general spatial congruence principle provides new evidence for the hypothesis that body ownership can be seen as a multisensory perception of one’s own body.

The second experiment showed that a relatively subtle tactile conflict between the seen and felt textures of the objects touching the rubber hand and the real hand significantly reduces the strength of the RHI. This observation suggests that the previously found lack of an effect of tactile incongruence on the RHI probably reflects the lack of sensitivity of the more traditional methods used to quantify the illusion (Schütz-Bosbach et al., 2009; Ward et al., 2015). Conceptually, our study is thus also important because it conclusively establishes tactile congruence of the seen and felt objects as a basic rule that determines the RHI. This tactile congruency rule is based on the multisensory congruence between the microgeometric features, such as texture (as shown in the present study), and the macrogeometric properties of the seen and felt objects touching the hands (e.g., shape; Ward et al., 2015), consistent with the “unity assumption” principle, which states that only meaningful combinations of crossmodal stimuli are integrated (De Gelder & Bertelson, 2003; Vatakis & Spence, 2007); for example, it has been found that congruent pairs of sounds and images of cats and dogs are integrated, in contrast to incongruent combinations of such stimuli presented at the same time and place (Hein et al., 2007). Thus, similar to the general principles of multisensory perception (Spence, 2011), body ownership is influenced not only by spatiotemporal correlations but also by congruencies in other stimulus dimensions.

### Future applications of the method in psychological science, cognitive psychiatry, and neuroscience

We are optimistic that our psychophysical discrimination task can be used in future work to address unresolved issues in the current literature on body ownership. For example, the task could be used to clarify the controversial issue of whether motor commands from active movement boost ownership over and above the somatosensory feedback signals in the so-called moving RHI (Dummer, Picot-Annand, Neal, & Moore, 2009; Kalckert & Ehrsson, 2012, 2014) or to examine whether fine-grained differences in the shape and “human-likeness” of the rubber hand affect the illusion beyond gross and categorical differences (Tsakiris et al., 2010), which a multisensory model would predict (based on visuoproprioceptive congruence). Our method could also be particularly valuable in cognitive psychiatry research for registering body ownership in individuals with disturbed or altered cognition that make their questionnaires ratings particularly unreliable and susceptible to confabulation and task compliance (e.g., people suffering from schizophrenia; Peled, Ritsner, Hirschmann, Geva, & Modai, 2000; Prikken et al., 2019), individuals receiving pharmacological intervention with psychoactive drugs (Morgan et al., 2011), or populations who are prone to psychosis (Germine, Benson, Cohen, & Hooker, 2013). Furthermore, based on unpublished observations, we are optimistic that our 2-AFC discrimination task could be extended to the whole body by using the full-body ownership illusion (Petkova & Ehrsson, 2008) with pairs of mannequins (Petkova et al., 2011), strangers’ bodies (Preston & Ehrsson, 2016), and computer-generated avatars (Maselli & Slater, 2013), which would enable rigorous and unbiased estimation of ownership percepts that encompass the entire body.

It should also be noted that the present behavioral results are consistent with neuroimaging studies that have demonstrated activity in frontal and parietal areas related to the integration of visual, tactile, and proprioceptive signals (Gentile, Guterstam, Brozzoli, & Ehrsson, 2013; Gentile, Petkova, & Ehrsson, 2011; Limanowski & Blankenburg, 2016) during the RHI (Ehrsson et al., 2004; Guterstam et al., 2019) and that the strength of this activation correlates with the strength of hand ownership as rated in questionnaires (Ehrsson et al., 2004; Gentile et al., 2013; Guterstam et al., 2013). Future fMRI studies could use the current psychophysical approach to examine whether the activity in these areas follows the same psychometric curves as the behavioral discriminations, and future transcranial magnetic stimulation studies could use the current approach as a sensitive method to test whether transient perturbation of the neural processing in these areas affects the perceptual ownership discriminations.

### Limitations

A few of limitations of the study deserves consideration. The first relate to the ecological validity of using body illusions—and especially the current “supernumerary RHI”—to investigate own-body perception, and the related concern that the results may say more about how illusions works rather than reveal something fundamental about body perception. We argue that veridical perception and illusory perception are essentially the same in that both arise as consequences of the way our perceptual systems process information. Thus, the classic RHI and the current version with two rubber hands probably involves the same multisensory binding mechanisms that operate for the real hand under everyday situations. The use of illusion to study body ownership is further motivated by the fact that it is not possible to manipulate body ownership by simply varying a single low-level sensory stimulus parameter (such as luminance for vision in classical psychophysics experiments), but the perceptual changes of interest happens as a consequence of the interpretation of the patterns of multisensory information at a level of processing above low-level unimodal sensation. A critical reader may still wonder whether experiencing a varying degree of ownership for two identical hands is not a bit odd, but earlier studies have shown that such experiences can readily be elicited in laboratory settings (Ehrsson, 2009; Guterstam et al., 2011; Newport et al., 2010), and our experience from the current study is that participants find this task naturally intuitive and easy to perform (“Which hand feels most like yours?”).

Another limitation of the study is that we only included participants who could experience the classic RHI. Thus, our results generalize to the approximately two-thirds of the population that can experience this body illusion (Kalckert & Ehrsson, 2014). We reasoned that the discriminations would turn into random guessing for individuals who did not have the capacity to experience the RHI—an assumption that was supported by pilot experiments conducted with such individuals (see Supplementary Figs. S8–S9). This deserves be further examined in future experiments to see if there is a systematic relationship between the susceptibility on the RHI across individuals in a sample that is representative for the entire population and the ability to discriminate between the two rubber hands in the present 2-AFC paradigm.

The putative concern that the participants might be “solving” the 2-AFC discrimination task, not by choosing rubber-hand-based illusory sense of ownership but based on visuotactile synchronicity, can be refuted based on four observations. First, the spatial manipulation in Experiment 1 should not affect the responses if the participants were basing their responses on visuotactile synchrony, but this manipulation changed the responses consistent with the RHI. Second, in the control experiment, the participants generated random responses across asynchronies in the two control conditions, which indicated that they were faithful to the task instructions and tried to discriminate ownership even though the RHI had been eliminated. Third, experiments conducted with participants who did not experience the classic RHI assessed in the initial screening test indicated that these individuals were not making responses based on visuotactile synchrony even though they were not able to discriminate ownership (see Supplementary Figs. S8–S9). Fourth, the tactile congruence effect we observed in Experiment 2 cannot be explained by synchronicity judgments. Thus, the current 2-AFC discrimination task registers body ownership perception in the RHI.

A final limitation worth mentioning is that although the results indicate that the current psychophysics task is more sensitive, robust, and less affected by cognitive biases than classic RHI measures, this assertion is based only on the impression that the present hand-ownership discriminations appeared to be affected by smaller temporal, spatial, and tactile incongruencies than questionnaires and the proprioceptive drift tests used in previously published studies. However, it is important to keep in mind that we have not directly compared the current discriminating paradigm with the classic tests, or any other alternative psychophysics approaches for that matter (e.g., detection task with one rubber hand), so no definite conclusions can be made regarding the relative sensitivity or robustness of the current method with respect to other methods. This methodological issue should be further examined in future studies.

### Conclusion

In summary, by developing the current psychophysical discrimination approach to the RHI, we were able to probe the spatial, temporal, and tactile congruency principles of body ownership at a finer scale and with better protection from cognitive bias than previous studies. The results provide empirical evidence in support of several important predictions from computational models of multisensory perception, and collectively suggest that the processes mediating the sense of ownership of one’s body are ruled by the same perceptual principles govern the multisensory perception of external objects. These findings support the hypothesis that body ownership can be defined as multisensory perception of one’s own body, which has important bearings on theories of body representation, bodily self, self-consciousness, disorders of bodily awareness, and prosthesis and avatar embodiment in the engineering sciences.

## Electronic supplementary material

ESM 1 (PDF 1390 kb)
