# Supplementary material for: Which hand is mine? Discriminating body ownership perception in a two-alternative forced-choice task
Source: Atten Percept Psychophys. 2020 Aug 27;82(8):4058–83. doi: 10.3758/s13414-020-02107-x (PMC7593318; doi:10.3758/s13414-020-02107-x)

Supplementary material

Inclusion phase:

Figure S.1: Additional picture of the robotic arms used in both experiments

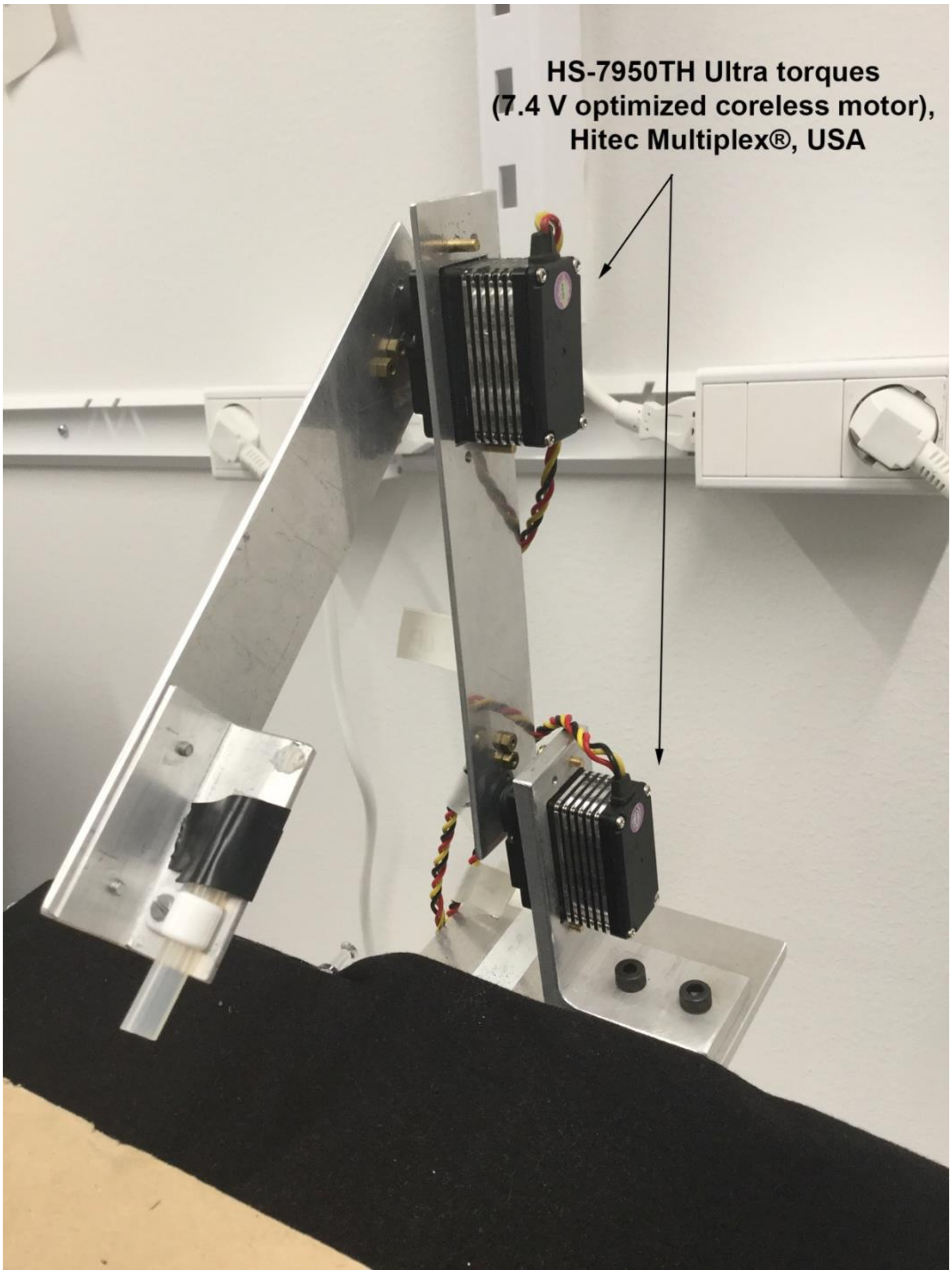

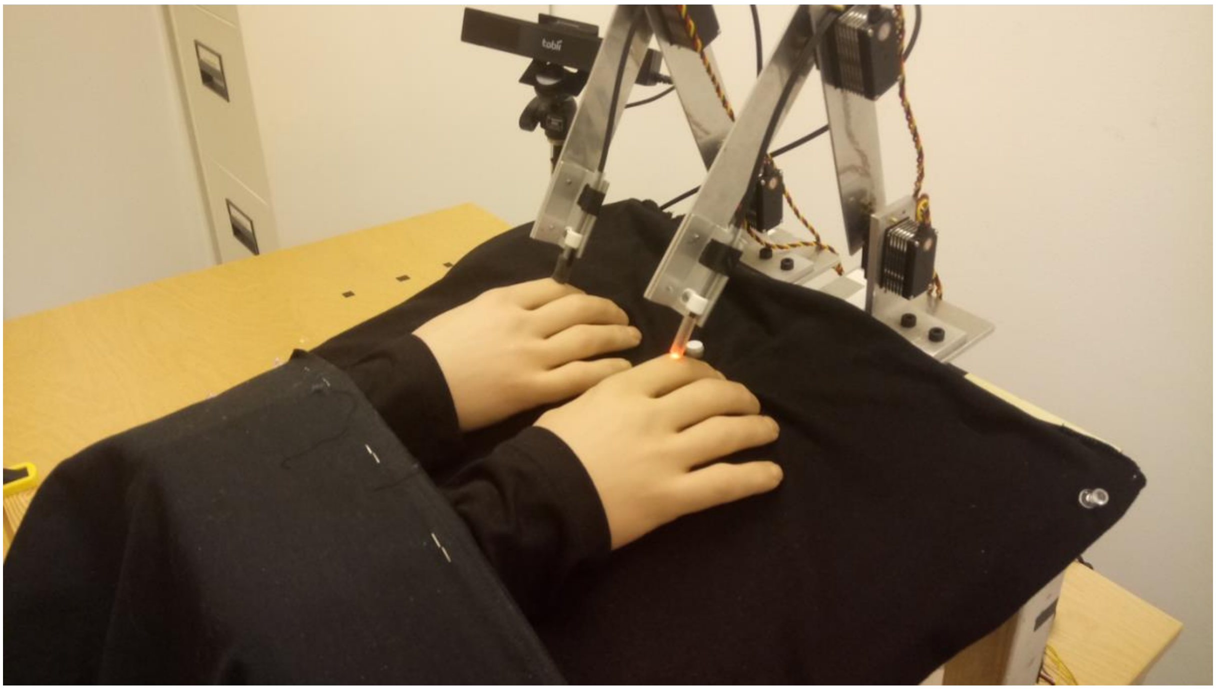

Figure S.2: Questionnaire items to screen individuals who did not experience the RHI during the inclusion phase.

Questionnaire

Rate the items according to what you felt (-3: I completely disagree; +3: I completely agree)

Q1. It seemed as if I were feeling the touch in the location where I saw the rubber hand touched.

-3	-2	-1	0	+1	+2	+3
☐	☐	☐	☐	☐	☐	☐

Q2. It seemed as though the touch I felt was caused by the stick touching the rubber hand

-3	-2	-1	0	+1	+2	+3
☐	☐	☐	☐	☐	☐	☐

Q3. I felt as if the rubber hand were my hand

-3	-2	-1	0	+1	+2	+3
☐	☐	☐	☐	☐	☐	☐

Q4. It felt as if my (real) hand were drifting towards up (towards the rubber hand).

-3	-2	-1	0	+1	+2	+3
☐	☐	☐	☐	☐	☐	☐

Q5. It seemed as if I might have more than one left hand or arm.

-3	-2	-1	0	+1	+2	+3
☐	☐	☐	☐	☐	☐	☐

Q6. It seemed as if the touch I was feeling came from somewhere between my own hand and the rubber hand

-3	-2	-1	0	+1	+2	+3
☐	☐	☐	☐	☐	☐	☐

Q7. It felt as if my (real) hand was turning 'rubbery'.

-3	-2	-1	0	+1	+2	+3
☐	☐	☐	☐	☐	☐	☐

Q8. It appeared (visually) as if the rubber hand were drifting towards my hand.

-3	-2	-1	0	+1	+2	+3
☐	☐	☐	☐	☐	☐	☐

Q9. The rubber hand began to resemble my own (real) hand, in terms of shape, skin tone, freckles or some other visual feature.

-3	-2	-1	0	+1	+2	+3
☐	☐	☐	☐	☐	☐	☐

Figure S.3: Questionnaire results for the participants included in experiment 1. The first three statements concern ownership perception, while the other 6 are control statements. Only the participants with a clear RHI experience (ownership score>1; difference ownership/control>1) were allowed to take part in experiment 1.

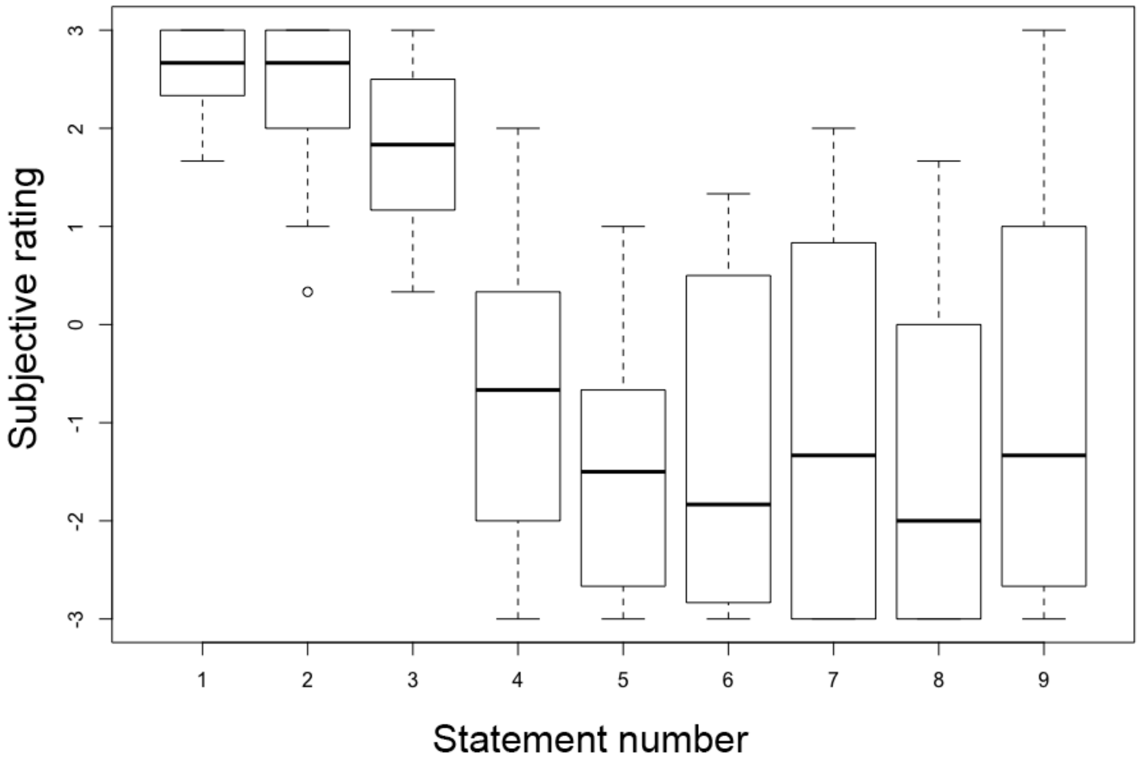

Figure S.4: Questionnaire results for the participants included in experiment 2. The first three statements concern ownership perception, while the other 6 are control statements. Only the participants with a clear RHI experience (ownership score>1; difference ownership/control>1) were allowed to take part in experiment 2.

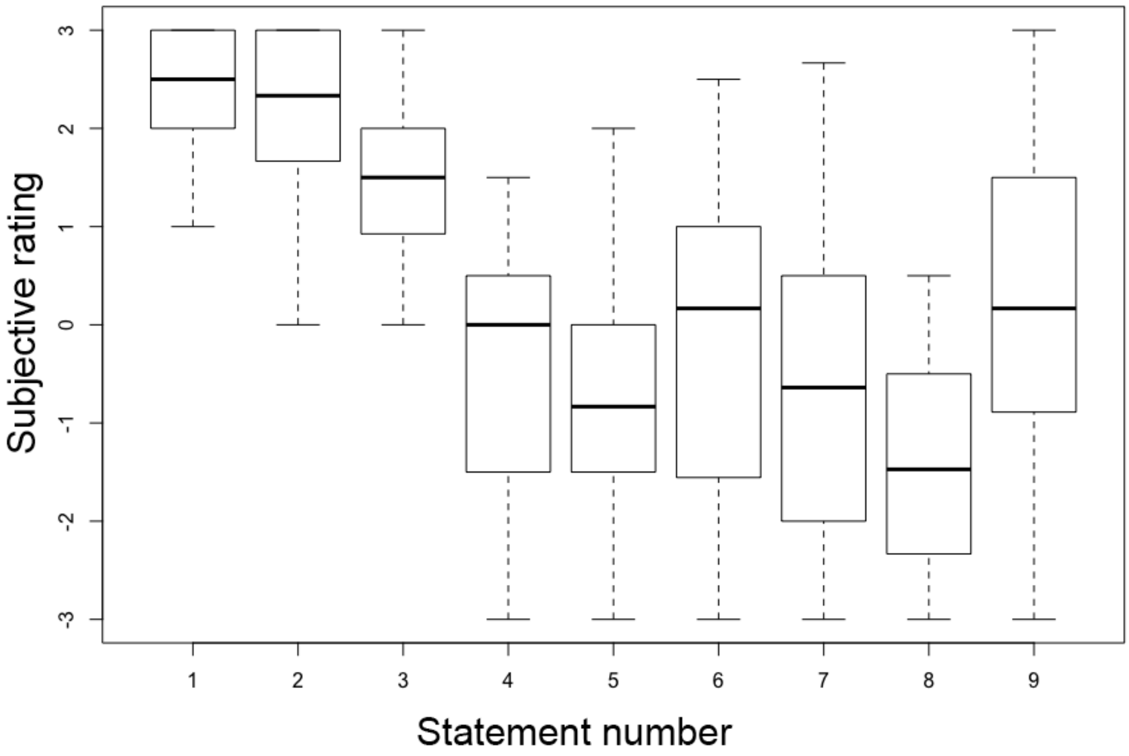

Table S.1: Individual results of the control experiment.
The values in red are those outside of the interval [mean – 3 x SD; mean + 3 x SD] calculated using the PSE and sigma values obtained in the *illusion* condition.

PSE	Illusion	Wider	Rotated	SIGMA	Illusion	Wider	Rotated
1	44	615	1840000	1	262	1190	10500000
2	141	43100	47500	2	440	1620000	245000000000000000000
3	-47	-55	-182	3	79	209000000000000000	464
4	-1	-1	-161	4	277	445000000000000000	630
5	-62	-483	-1	5	160	74760	1151
6	-62	-1057	0	6	160	2871	911
7	-55	-226	-4370	7	129	903	11100
8	-49	-62	-402	8	176	40050000000000000	4600
9	-37	-43	127	9	241	1320000000000000000	1980
10	-18	-219	-194	10	140	1080	963

Mean	-15	Mean	206
SD	61	SD	98
Mean + 3 SD	167	Mean + 3 SD	500
Mean - 3 SD	-196	Mean - 3 SD	-87

Figure S.5: Plots for individual subjects in experiment 1

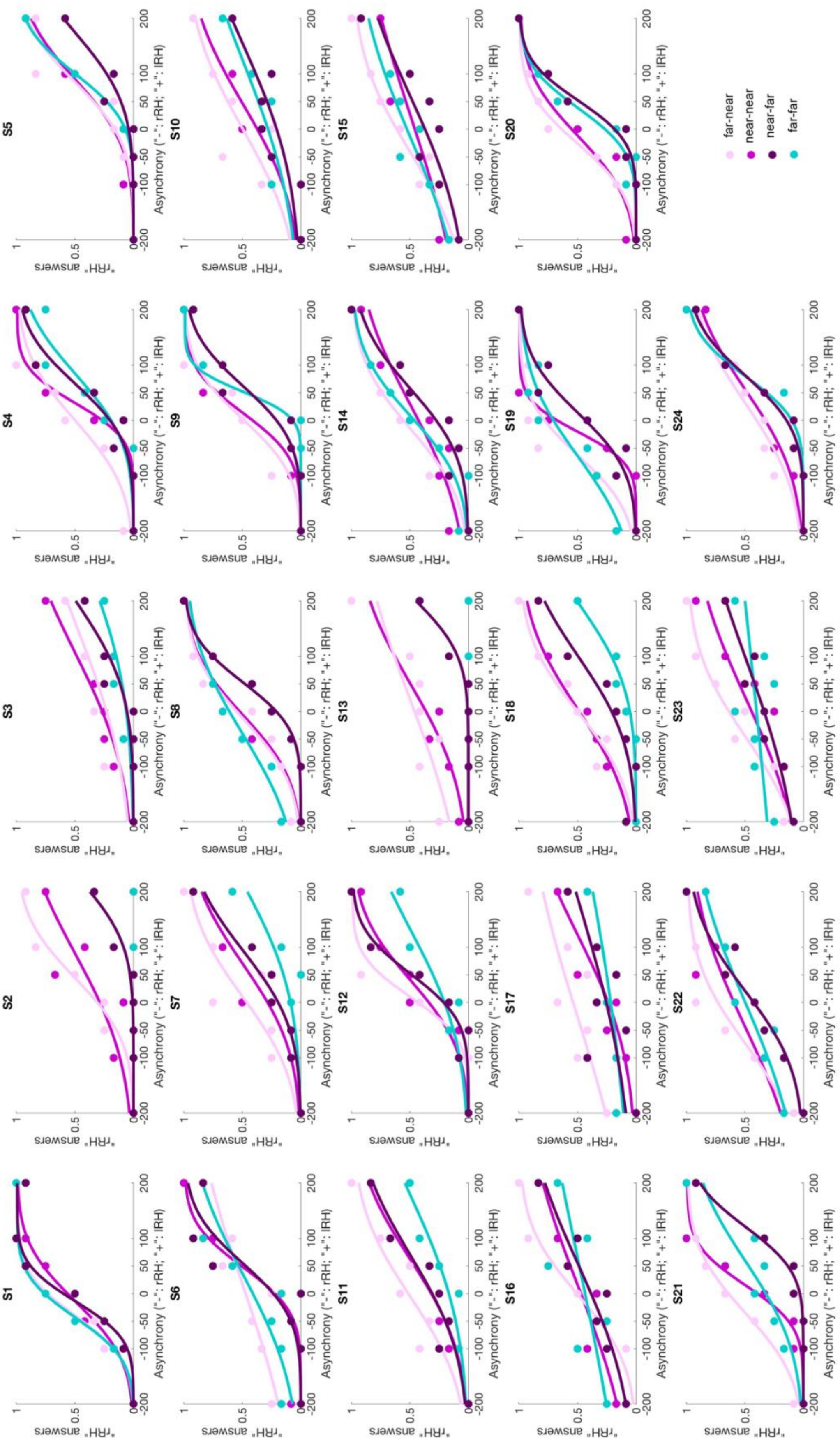

Figure S.6: Plots for individual subjects in experiment 2 when the real hand is touched by plastic.

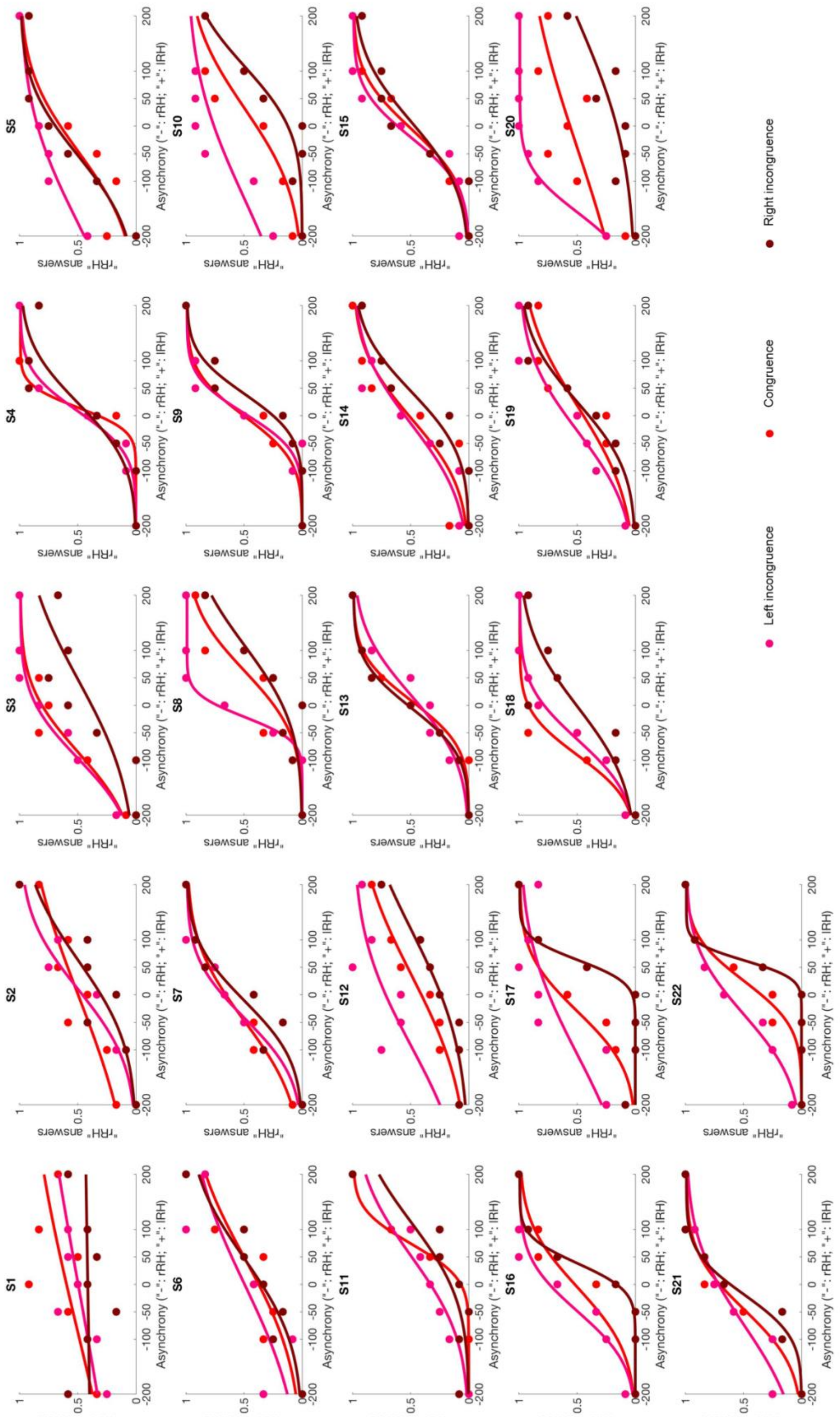

Figure S.7: Plots for individual subjects in experiment 2 when the real hand is touched by foam

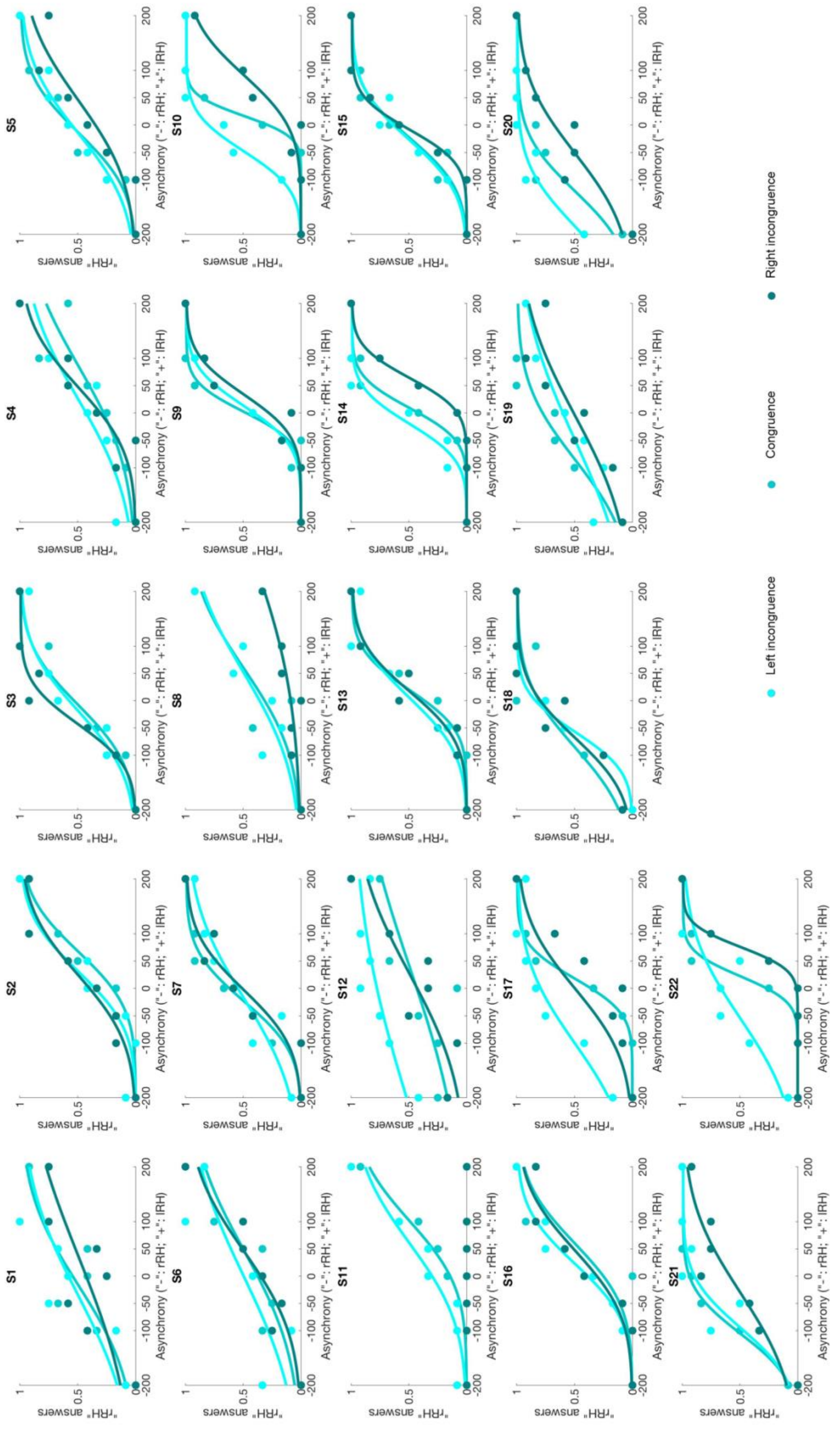

Figure S.8: The proportion of "the right rubber hand felt the most like my hand" answers under different asynchrony conditions in participants who did not feel the RHI.

These 15 participants (8 males, 26.3 +/- 4 years) did not pass the inclusion criterion when tested on the RHI in the screening procedure but were tested in the 2-AFC ownership discrimination task. The same procedure was used as in the main experiment but with only one condition, namely, that when the two rubber hands were placed at identical distances from the real hand and touched by the firm plastic tubes at the seven asynchronies (12 seconds of visual tactile stimulation, 12 repetitions per condition, one experimental block). However, there were some slight variations in the setups as these participants were tested in different experiments (S1: the rubber hands were 6 cm away from the real hand instead of 5 cm; S2 to S5: experiment 1 setup; S6 to S9: experiment 2 setup; S10 to S15: setup included small changes in the robots and table design for another study conducted by our team), but these differences were small and should not have affected the basic discrimination task. As expected, these individuals were unable to reliably discriminate illusory hand ownership. Specifically, the results suggest that 14 out of the 15 participants were unable to discriminate illusory ownership because their data did not fit the cumulative Gaussian function and/or the corresponding fitting parameters were behaviorally nonsensical (e.g., a PSE equal to 571 ms, which indicated that the rubber hands were as likely to be chosen by the participant when the right rubber hand was touched synchronously with the real hand and the left rubber hand 571 ms later). These patterns of responses were different from the groups of illusion responders in the main experiments (compare to Fig. S5, S6, S7) but similar to those observed in the control experiment under the condition where body ownership towards any rubber hand was not elicited. These results are also informative in that they show that these individuals followed the original instruction and did not try to overcome the lack of illusion by switching strategies and starting to report visuotactile synchrony instead.

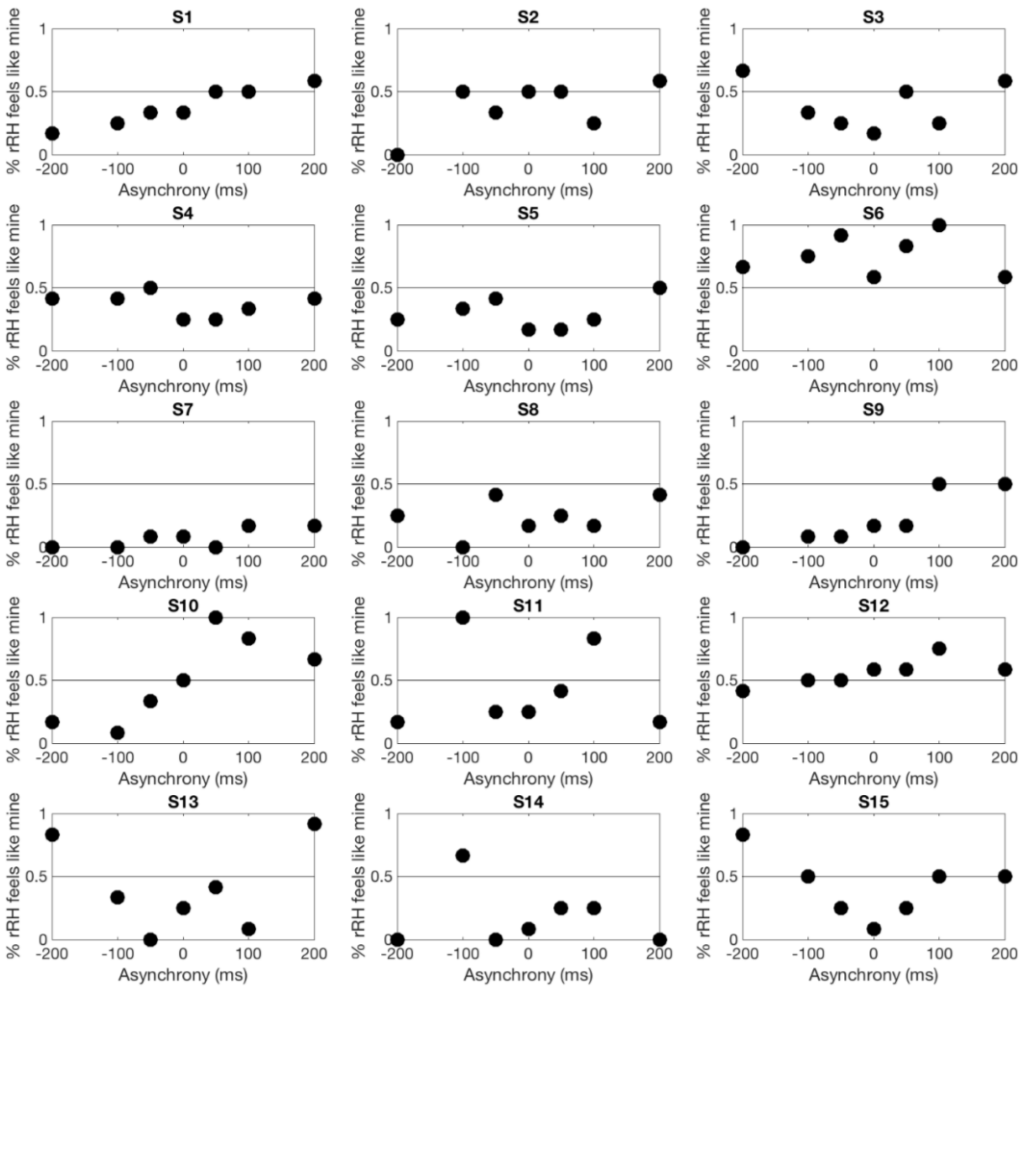

Figure S.9: Number of “the right rubber hand felt the most like my hand” answers under different asynchrony conditions for 15 participants who did not feel the RHI. These data are plotted for descriptive purposes only.

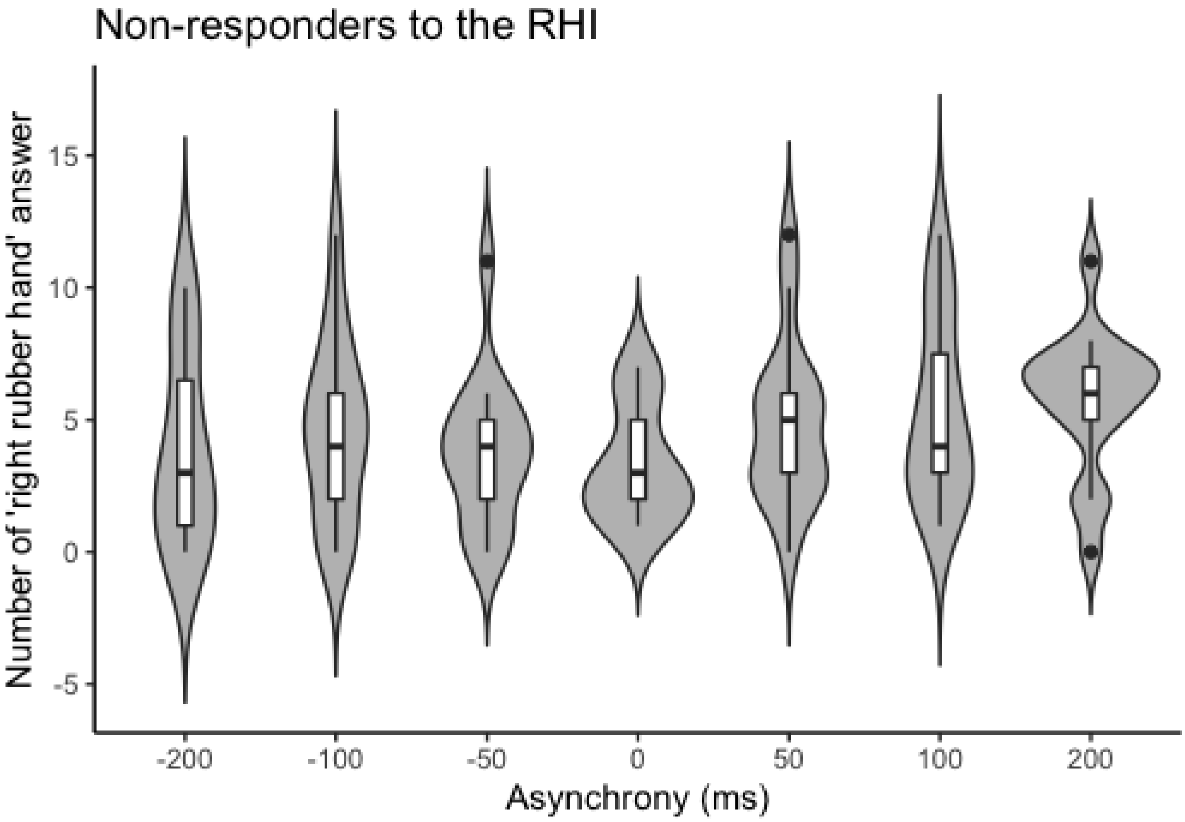

Supplement: Supplementary file 1 — (PDF 1390 kb) [file 13414_2020_2107_MOESM1_ESM.pdf]
